# Reshaping the chromatin landscape in HUVECs from small-for-gestational-age newborns

**DOI:** 10.1172/jci.insight.186812

**Published:** 2025-04-22

**Authors:** Lingling Yan, Zhimin Zhou, Shengcai Chen, Xin Feng, Junwen Mao, Fang Luo, Jianfang Zhu, Xiuying Chen, Yingying Hu, Yuan Wang, Bingbing Wu, Lizhong Du, Chunlin Wang, Liang Gong, Yanfen Zhu

**Affiliations:** 1 Department of Pediatrics, The First Affiliated Hospital, Zhejiang University School of Medicine, Hangzhou, China.; 2 Center for Regeneration and Aging and; 3 Department of Obstetrics and Gynecology, Center for Reproductive Medicine, the Fourth Affiliated Hospital of School of Medicine, and International School of Medicine, International Institutes of Medicine, Zhejiang University, Yiwu, China.; 4 Department of Pediatrics, Children’s Hospital of Zhejiang University School of Medicine, Hangzhou, China.; 5 Liangzhu Laboratory, Zhejiang University, Hangzhou, China.

**Keywords:** Angiogenesis, Cardiology, Epigenetics

## Abstract

Small for gestational age (SGA), with increased risk of adult-onset cardiovascular diseases and metabolic syndromes, is known to associate with endothelial dysfunction, but the pathogenic mechanisms remain unclear. In this study, the pathological state of human umbilical vein endothelial cells (HUVECs) from SGA individuals was characterized by presenting increased angiogenesis, migration, proliferation, and wound healing ability relative to their normal counterparts. Genome-wide mapping of transcriptomes and open chromatins unveiled global gene expression alterations and chromatin remodeling in SGA-HUVECs. Specifically, we revealed increased chromatin accessibility at active enhancers, along with dysregulation of genes associated with angiogenesis, and further identified *CD44* as the key gene driving HUVECs’ dysfunction by regulating pro-angiogenic genes’ expression and activating phosphorylated ERK1/2 and phosphorylated endothelial NOS expression in SGA. In SGA-HUVECs, *CD44* was abnormally upregulated by 3 active enhancers that displayed increased chromatin accessibility and interacted with *CD44* promoter. Subsequent motif analysis uncovered activating protein-1 (AP-1) as a crucial transcription factor regulating *CD44* expression by binding to *CD44* promoter and associated enhancers. Enhancers CRISPR interference and AP-1 inhibition restored *CD44* expression and alleviated the hyperangiogenesis of SGA-HUVECs. Together, our study provides a foundational understanding of the epigenetic alterations driving pathological angiogenesis and offers potential therapeutic insights into addressing endothelial dysfunction in SGA.

## Abstract 2

Small for gestational age (SGA) individuals show abnormal blood vessel growth due to epigeneitic changes, specifically CD44, which could lead to adult onset diseases.

## Graphical abstract

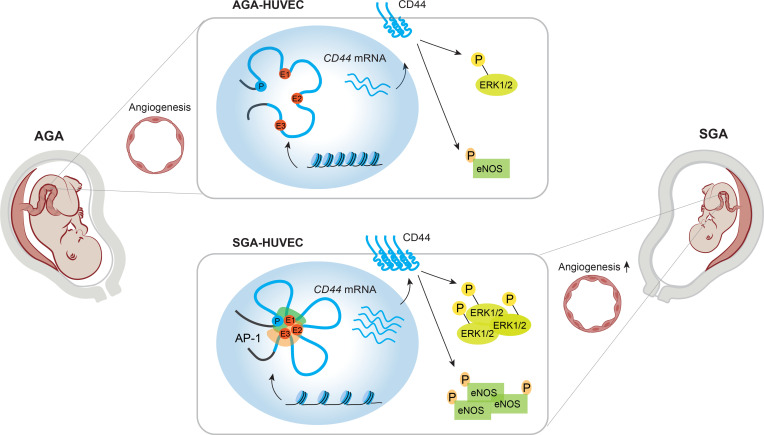

## Introduction

Small for gestational age (SGA) describes infants with birth weights below the 10th percentile compared with their appropriate-for-gestational-age (AGA) counterparts (1). Advances in perinatal monitoring and management, along with neonatal resuscitation, have significantly improved survival rates for SGA infants. However, these individuals remain at heightened risk for adverse vascular outcomes, including impaired vasodilatation (2), fetal cardiovascular programming (3), and increased arterial wall thickness (4, 5), ultimately predisposing them to adult-onset cardiovascular and metabolic diseases, particularly in the presence of genetic susceptibilities (6, 7). Endothelial cells, which form the inner lining of blood vessels, serve as a dynamic interface between the circulatory system and surrounding tissues, critically regulating vascular tone and cellular functions (8). Impaired endothelial function is closely related to the pathological vascular processes in cardiovascular diseases and diabetic vascular complications (9). Therefore, preserving endothelial function during early life stages is crucial for maintaining vascular health over the long term. Impairments in early angiogenesis can contribute to lifelong vascular dysfunction, making endothelial cells a compelling therapeutic target for mitigating SGA-associated cardiovascular and metabolic risks in adulthood.

Human umbilical vein endothelial cells (HUVECs), which bridge the maternal and fetal circulation, play a pivotal role in fetal growth and development by regulating the formation and function of the fetal vascular system (10). Emerging evidence highlights significant differences in the angiogenic capacity of HUVECs derived from SGA neonates compared with their AGA counterparts (11, 12). These disparities are accompanied by distinct protein expression profiles and DNA methylation patterns (9, 10, 13). Furthermore, epigenetic modifications have been implicated in the transgenerational inheritance of impaired vascular function, as observed in studies on SGA rats (14, 15). Despite these insights, the precise contributions of epigenetic factors to HUVEC dysfunction in SGA and the underlying mechanisms remain largely unresolved. Given the critical role of HUVECs in fetal vascular biology and their accessibility from umbilical cords, primary cultured HUVECs offer a robust model for investigating fetal endothelial dysfunction and the developmental origins of adult cardiovascular and metabolic diseases in SGA (10, 16–18).

CD44, a cell surface glycoprotein widely expressed on mammalian cell surfaces, including endothelial cells, epithelial cells, fibroblasts, and white blood cells (19), plays essential roles in cell-cell interactions, cell adhesion, cell proliferation, and migration (20). Evidence from *Cd44*-knockout mice further supports its potential role in both physiological vascular development and pathological vascular dysfunction (21, 22). Here, we characterized the dysfunction of HUVECs derived from SGA status and examined the landscape of chromatin openness by performing RNA-Seq and assay for transposase-accessible chromatin with high-throughput sequencing (ATAC-Seq). We identified *CD44* as the major causal gene driving the expression of pro-angiogenic genes and activating phosphorylated ERK1/2 and phosphorylated endothelial NOS (p-ERK1/2 and p-eNOS) expression in SGA and further elucidated aberrant accessible enhancers targeted by activating protein-1 (AP-1) transcription factors, resulting in upregulated *CD44* expression and enhanced angiogenic potential in SGA-HUVECs. These findings suggest that the epigenetic regulation of *CD44* may be crucial in determining endothelial function and angiogenesis in SGA individuals and could influence the development and progression of fetal-origin, adult-onset diseases.

## Results

### Promoted proliferation, migration, and angiogenesis of HUVECs in SGA.

**Figure 1:**
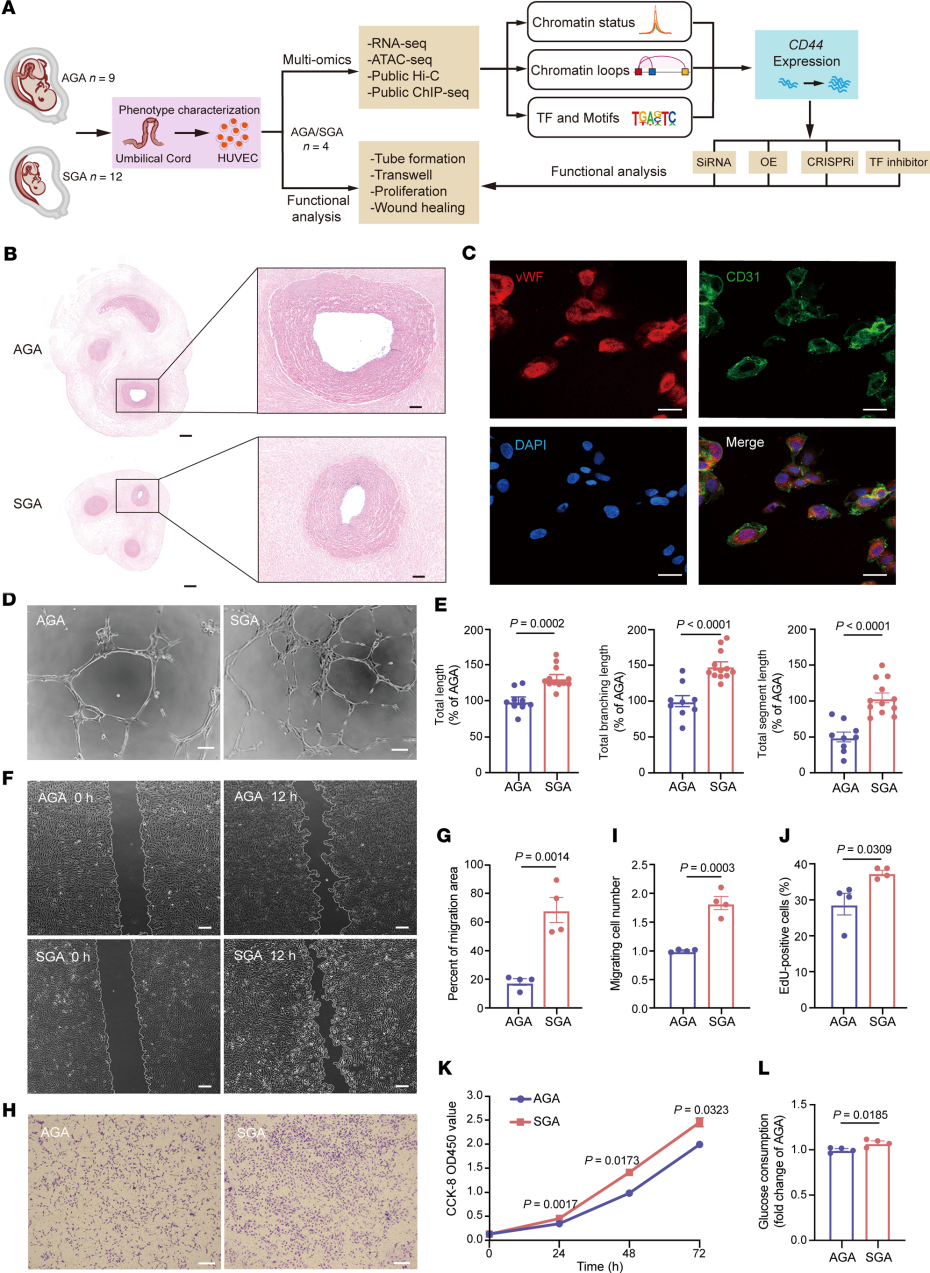
Distinctive phenotypes of HUVECs derived from SGA and AGA individuals. (**A**) Schematic overview of the study design. CRISPRi, CRISPR interference; Hi-C, high-throughput chromosome conformation capture; OE, overexpression; TF, transcription factor. (**B**) Representative images of the cross section of umbilical cord tissues stained by H&E. The enlarged parts represent umbilical veins. Scale bars: 1 mm (left) and 200 μm (right); *n* = 4 per group. (**C**) Representative immunofluorescence staining of primary HUVECs’ identity; scale bar: 20 μm; *n* = 4 per group. (**D**) Representative images of angiogenesis; scale bar: 100 μm. (**E**) Analysis of the total tube length, total tube branching length, and total segment length for samples shown in Supplemental Figure 1A (*n* = 9 AGA and 12 SGA). (**F**) Representative images of scratches at 0 hours and 12 hours; scale bar: 200 μm. (**G**) Analysis of the percentage of migration area; *n* = 4 per group. (**H**) Representative images of HUVECs that migrated through the pores; scale bar: 100 μm. (**I**) Analysis of percentage of migrating cell number; *n* = 4 per group. (**J**) Analysis of EdU assay; *n* = 4 per group. (**K**) Analysis of CCK8 assay results at 0 hours, 24 hours, 48 hours, and 72 hours; *n* = 4 per group. Two-way ANOVA with Holm-Šídák multiple comparisons test was used for comparing cell proliferation at different times. (**L**) Analysis of glucose consumption; *n* = 4 per group. Data presented as mean ± SEM. Statistical analysis was performed using 2-tailed unpaired Student’s *t* test.

To examine the epigenetic changes associated with SGA status, we conducted comprehensive analysis as shown in Figure 1A. We collected umbilical cord samples from 12 SGA neonates diagnosed by birth weight below 10th percentile for gestational age as well as 9 AGA neonates with birth weight ranging from 10th to 90th percentiles as a control group following the criteria outlined in China (23). To minimize the impact of maternal factors, all specimens were obtained exclusively from full-term individuals without medical or obstetrical complications, except for 1 SGA mother who experienced preeclampsia during pregnancy. Compared with AGA, the SGA group exhibited significant reductions in birth weight (SGA: 2.15 ± 0.16 kg; AGA: 3.47 ± 0.12 kg; *P* < 0.0001), birth length (SGA: 46.08 ± 0.76 cm; AGA: 49.67 ± 0.47 cm; *P* = 0.0020), and maternal BMI at delivery (*P* = 0.0130) but nonsignificant changes in maternal age, gestational age, and pre-pregnancy BMI, as detailed in Supplemental Tables 1 and 2; supplemental material available online with this article; https://doi.org/10.1172/jci.insight.186812DS1 In addition, a notably smaller diameter of both umbilical cord and umbilical vein in the SGA group was observed (Figure 1B), suggesting potential abnormalities in HUVECs’ functions in SGA neonates.

To further investigate the influence of SGA status on HUVECs’ function, we successfully isolated and cultured the primary HUVECs from both SGA and AGA groups in vitro and confirmed their identity by the characteristic cobblestone cell shapes and high expression of endothelial markers vWF and CD31 (Figure 1C). Interestingly, relative to AGA-HUVECs, SGA-HUVECs exhibited increased in vitro angiogenic capacity with significantly increased total length (*P* = 0.0002), total branching length (*P* < 0.0001), and total segment length (*P* < 0.0001) of tubes in the tube formation assay (Figure 1, D and E, and Supplemental Figure 1A). Meanwhile, promoted migration and invasive ability were observed in SGA-HUVECs, as evidenced, respectively, by a significantly larger migration area 12 hours after injury in the scratch wound healing assay (Figure 1, F and G, *P* = 0.0014) and by a significantly increased number of cells migrating through the pores in the Transwell assay (Figure 1, H and I, *P* = 0.0003) relative to AGA-HUVECs. Assessment of proliferation ability by 5-ethynyl-2′-deoxyuridine (EdU) nucleic acid labeling technique and cell counting kit-8 (CCK8) assays revealed a significantly higher proliferation rate (*P* = 0.0309 in EdU assay and *P* = 0.0017 at 24 hours in CCK8 assay) in SGA-HUVECs compared with AGA-HUVECs (Figure 1, J and K). Consistent with their increased cellular activity, SGA-HUVECs displayed a higher consumption of glucose compared with the AGA group after 24 hours of culture, indicating an elevated energy requirement to support their improved survival (Figure 1L, *P* = 0.0185). There was also an elevated reactive oxygen species (ROS) generation in SGA-HUVECs compared with AGA (Supplemental Figure 1B).

Taken together, our results suggest that the SGA condition may hinder umbilical cord development, particularly affecting the formation of the umbilical vein and the function of HUVECs. Specifically, the SGA state exerts strong stimulatory effects on the angiogenesis, migration, proliferation, and wound healing capacity of HUVECs.

### Dysregulation of angiogenic genes in SGA-HUVECs.

**Figure 2:**
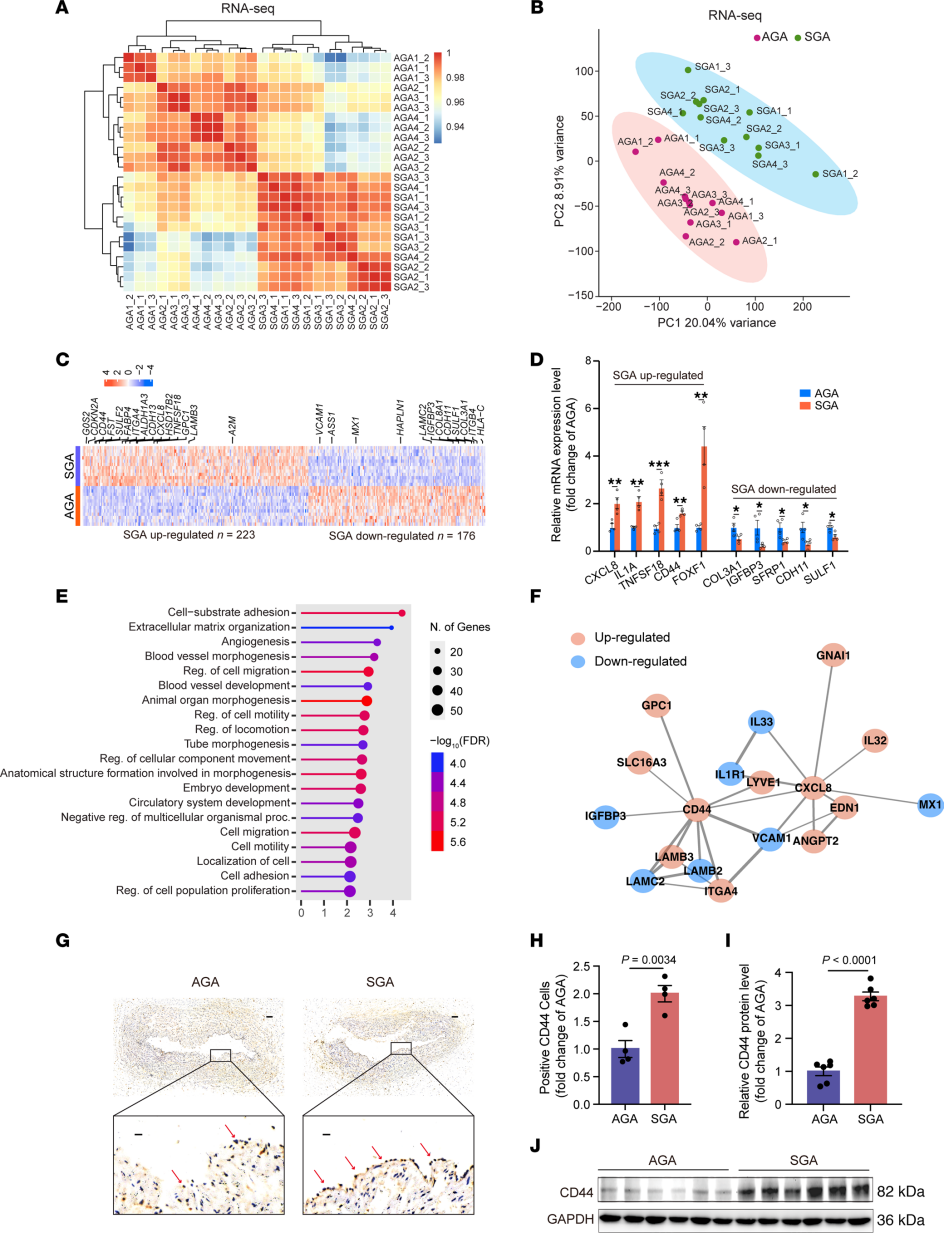
Dysregulation of angiogenic genes in SGA-HUVECs. (**A**) Clustering of RNA-Seq data by correlation of fragments per kilobase million (FPKM) between samples. (**B**) PCA of RNA-Seq data based on FPKM values. (**C**) Heatmap of the normalized expression of the 399 DEGs in SGA relative to AGA. Selected genes are labeled. Red color indicates upregulation and blue color indicates downregulation. (**D**) Validation of RNA-Seq data by RT-qPCR. Relative expression of 10 selected genes in 4 biological replicates were displayed. *P ≤ 0.05, **P ≤ 0.01, ***P ≤ 0.001. (**E**) The top 20 significantly enriched GO terms of biological process in DEGs with the FDR < 0.05. (**F**) PPI subnetwork with CD44 as hub nodes. First neighbors of hub nodes were shown. The thicker the edge, the higher the combined score between 2 nodes. (**G**) Representative IHC images of paraffin-embedded umbilical cord samples using CD44 antibody. Arrows indicate HUVECs with high CD44 expression; scale bars: 200 μm (upper) and 25 μm (lower). (**H**) Analysis of percentage of CD44-positive cells in intima of umbilical veins from AGA and SGA; *n* = 4 per group. (**I** and **J**) Analysis of Western blot showing the high CD44 protein levels in HUVECs from SGA relative to AGA; *n* = 6 per group. The data in **D**, **H**, and **I** are presented as mean ± SEM and were analyzed using 2-tailed unpaired Student’s *t* test.

To further explore the mechanisms underlying the impaired SGA-HUVEC function, we performed RNA-Seq on 4 SGA-HUVECs and 4 AGA-HUVECs with 3 technical replicates. Both hierarchical clustering and principal component analysis (PCA) separated the SGA and AGA groups well (Figure 2, A and B), indicating a distinct gene regulation pattern between them. A total of 399 differentially expressed genes (DEGs) were identified in SGA relative to AGA with fold-change ≥ 1.50 and adjusted *P* value < 0.05, 223 of which were upregulated and 176 downregulated (Figure 2C and Supplemental Figure 2A). Real-time quantitative PCR (RT-qPCR) assay verified the upregulation of *CXCL8*, *IL1A*, *TNFSF18*, *CD44*, and *FOXF1* and the downregulation of *COL3A1*, *IGFBP3*, *SFRP1*, *CDH11*, and *SULF1* in SGA-HUVECs (Figure 2D). Among them, CD44 is a major cell surface receptor of hyaluronic acid that plays an essential role in physiological activities, including cell proliferation, adhesion, angiogenesis, and migration (24). *CXCL8*, also known as *IL-8*, promotes angiogenesis during wound healing, tissue repair, and cancer progression (25). Reduction of *CXCL8* expression attenuated tumor-associated angiogenesis (26). *SULF1* is a known antiangiogenic gene in multiple processes (27). To understand the functional association of the 399 DEGs, we performed gene ontology (GO) enrichment analysis (Figure 2E). We observed the enrichment of processes associated with development (embryo development, circulatory system development, and anatomical structure formation involved in morphogenesis), indicating low birth weight in SGA may disturb the impaired HUVECs’ function. Corresponding to the pro-angiogenic state of SGA-HUVECs, GO terms related to angiogenesis (blood vessel morphogenesis, blood vessel development, and tube morphogenesis), and extracellular matrix (cell-substrate adhesion and extracellular matrix organization), a structure playing significant roles in blood vessel formation and signaling (28), were enriched. Moreover, Kyoto Encyclopedia of Genes and Genomes analysis also identified ECM-receptor interaction as the top significant term (data not shown), indicating ECM-associated receptor and ligand proteins play a role here. These analyses indicated that genes associated with angiogenesis were dysregulated in SGA-HUVECs, which may result in impaired function of umbilical veins.

As 17.20% of protein-coding DEGs encoded receptor or ligand proteins (i.e., CD44, CXCL8, SULF1, LAMB2, IGFBP3, ANGPT2, and ITGB4), twice the proportion (8.00%) found in genome-wide protein-coding genes (29), we thus performed Protein-Protein Interaction (PPI) analysis of DEGs using the STRING database (30). A PPI network consisted of 105 nodes and 108 edges, with confidence scores ≥ 0.7 detected (Supplemental Figure 2B). CD44 was the top hub node with 10 first-level connected proteins, involving proteins functioning in growth (i.e., IGFBP3) (31, 32), angiogenesis, and vessel development (i.e., VCAM1 and LYVE1) (33, 34) (Figure 2F). Next, we verified the substantial increase of CD44 in the endothelial cells of umbilical cord tissues by immunohistochemistry (IHC) (Figure 2, G and H, and Supplemental Figure 3A). ELISA for CD44 in umbilical cord blood serum revealed a slight elevation of CD44 levels though without statistical significance in SGA compared with the AGA group (Supplemental Figure 3B). We further verified the elevated CD44 protein levels in SGA-HUVECs by Western blot compared with AGA-HUVECs (Figure 2, I and J). Inspired by the highly differential *CD44* expression and the key role of *CD44* in ECM signaling and angiogenesis, we speculated that *CD44* may function as the primary molecular determinant causing SGA-HUVECs’ dysfunction.

### CD44 as a key regulator strengthening angiogenesis of SGA-HUVECs.

**Figure 3:**
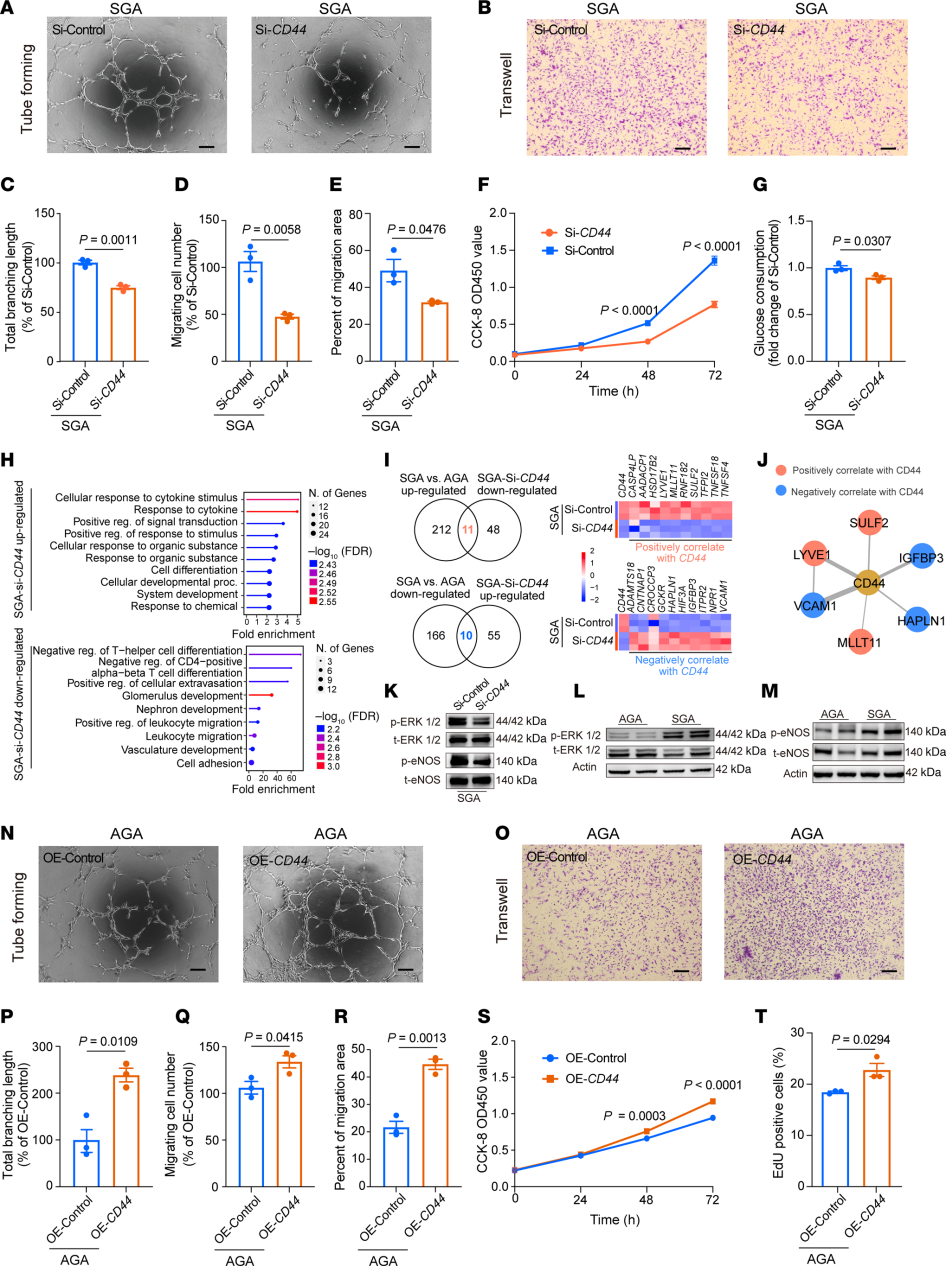
CD44 is a key regulator strengthening functions of SGA-HUVECs. (**A**) Representative tube formation images; scale bar: 100 μm. (**B**) Representative images of migrated cells; scale bar: 100 μm. (**C**) Analysis of the total tube branching length. (**D**) Percentage of migrating cell number. (**E**) Analysis of the percentage of migration area. (**F**) Analysis of CCK8 assay results at 0 hours, 24 hours, 48 hours, and 72 hours; 2-way ANOVA with Holm-Šídák multiple comparisons test was used; *n* = 3. (**G**) Analysis of glucose consumption. (**H**) Biological processes enriched in DEGs up- or downregulated in SGA-Si-*CD44* HUVECs. (**I**) Identification of genes positively or negatively correlated with *CD44* expression. The Venn diagram on the left shows the overlap of DEGs between SGA vs. AGA and SGA-si*CD44* vs. SGA-siControl. The right panel displays the relative expression of the overlapping DEGs. (**J**) PPI analysis of the proteins correlate with CD44 after CD44 knockdown in SGA-HUVECs. (**K**) Representative Western blot images of p-ERK1/2, total (t-) ERK1/2, p-eNOS, and t-eNOS in Si-Control and Si-*CD44* SGA-HUVECs; *n* = 3. (**L** and **M**) Representative Western blot images of p-ERK1/2, t-ERK1/2, p-eNOS, and t-eNOS in AGA- and SGA-HUVECs; *n* = 3–4. (**N**) Representative tube formation images; scale bar: 100 μm. (**O**) Representative images of migrated cells; scale bar: 100 μm. (**P**) Analysis of the total tube branching length. (**Q**) Percentage of migrating cell number. (**R**) Analysis of the percentage of migration area. (**S**) Analysis of CCK8 assay results at 0 hours, 24 hours, 48 hours, and 72 hours; 2-way ANOVA with Holm-Šídák multiple comparisons test was used; *n* = 3. (**T**) Analysis of EdU assay result of AGA-HUVECs with *CD44* overexpression. Data in **C**–**E**, **G**, **P**–**R**, and **T** are presented as mean ± SEM and were analyzed by 2-tailed unpaired Student’s *t* test; *n* = 3.

To validate our hypothesis, we employed small interfering RNA (siRNA) to specifically decrease *CD44* expression in the SGA condition. Following transfection with *CD44* siRNA (Si-*CD44*), *CD44* mRNA and protein levels were reduced by approximately 76.00% and 80.00%, respectively (Supplemental Figure 4, A–C). In SGA-HUVECs, diminished *CD44* expression led to reduced tube-forming ability (Figure 3A), marked by decreased total branching length (*P* = 0.0011), total segment length (*P* = 0.0060), and total length (*P* = 0.0011) of tubes (Figure 3C and Supplemental Figure 4, D and E), which is accordant with the previously reported results in *Cd44*-deficient mice that endothelial *Cd44* led to reduced vascularization and angiogenesis together with impaired vascular integrity (22, 35). Furthermore, reduced migration (Figure 3, B and D, *P* = 0.0058), wound healing capacity (Figure 3E and Supplemental Figure 4F, *P* = 0.0476), proliferation rate (Figure 3F and Supplemental Figure 4G, *P* < 0.0001 in CCK8 assay and *P* = 0.0026 in EdU assay), and glucose consumption (Figure 3G, *P* = 0.0307) were also observed in SGA-HUVECs when *CD44* was knocked down. These results underscore the key role of *CD44* in the pathogenesis of SGA-HUVECs and suggest that its reversion may offer a promising approach to restore balanced functions of SGA-HUVECs. Next, RNA-Seq analysis identified 65 upregulated DEGs, primarily enriched in cytokine-related pathways, and 59 downregulated DEGs, enriched in vasculature development, following *CD44* knockdown (Figure 3H). By overlapping SGA Si-*CD44* downregulated DEGs with those upregulated in SGA compared with AGA, we identified 11 key genes positively correlated with *CD44* expression, including the pro-angiogenic genes *LYVE1*, *SULF2*, and *TFPI2*. Similarly, by overlapping SGA Si-*CD44* upregulated DEGs with those downregulated in SGA compared with AGA, we identified 10 key genes negatively correlated with *CD44* expression, such as the inflammatory and fibrotic genes *VCAM1*, *IGFBP3*, *ADAMTS18*, and *HAPLN1* (Figure 3I). PPI analysis demonstrated that VCAM1, LYVE1, SULF2, IGFBP3, HAPLN1, and MLLT11 had direct protein interactions with CD44 (Figure 3J and Supplemental Table 3), indicating a complex regulatory network associated with CD44 protein. Since ERK1/2 is a key component of the mitogen-activated protein kinase (MAPK) pathway in angiogenesis (36), we investigated its phosphorylation in relation to *CD44* expression. Following *CD44* knockdown in SGA-HUVECs, we observed a significant reduction in p-ERK1/2 compared with the SGA Si-Control group (Figure 3K). Similarly, p-eNOS at Ser1177, a critical mediator of vascular homeostasis and angiogenesis (36), was also reduced after *CD44* knockdown (Figure 3K). Importantly, both p-ERK1/2 and p-eNOS levels were elevated in primary SGA-HUVECs compared with AGA-HUVECs (Figure 3, L and M, and Supplemental Figure 4, H and I), consistent with the higher *CD44* expression levels in SGA-HUVECs. These findings suggest that *CD44* promotes angiogenesis by regulating pro-angiogenic gene expression and promoting angiogenic signaling via the p-ERK1/2 and p-eNOS activation.

Meanwhile, we also evaluated whether overexpressing *CD44* could strengthen the abilities in AGA-HUVECs mentioned above. With nearly 4,000 times elevation at the mRNA level and 4.4 times increase at the protein level after *CD44* overexpression (Supplemental Figure 4, J–L), our results revealed an enhanced tube-forming ability in AGA-HUVECs upon *CD44* overexpression (Figure 3N), which was characterized by increased total branching length (*P* = 0.0109), total segment length (*P* = 0.0141), and total length (*P* = 0.0137) (Figure 3P and Supplemental Figure 4, M and N). Furthermore, enhanced migration (Figure 3, O and Q, *P* = 0.0415), wound healing capacity (Figure 3R and Supplemental Figure 4O, *P* = 0.0013), and proliferation rate (Figure 3, S and T, *P* < 0.0001 in CCK8 assay and *P* = 0.0294 in EdU assay) were also observed in AGA-HUVECs when *CD44* was overexpressed. Contrary to the results of *CD44* knockdown, overexpression of *CD44* in AGA-HUVECs significantly upregulated cell tube-forming ability and other phenotypes, further verifying the regulatory role of *CD44* in HUVEC dysfunction.

In conclusion, our comprehensive analysis highlights CD44 as a key molecular determinant in the dysfunction of SGA-HUVECs, whereas the specific mechanism governing the effects of elevated *CD44* expression on angiogenic ability awaits further elucidation.

### Genome-wide aberrant chromatin accessibility at enhancers in SGA-HUVECs.

**Figure 4:**
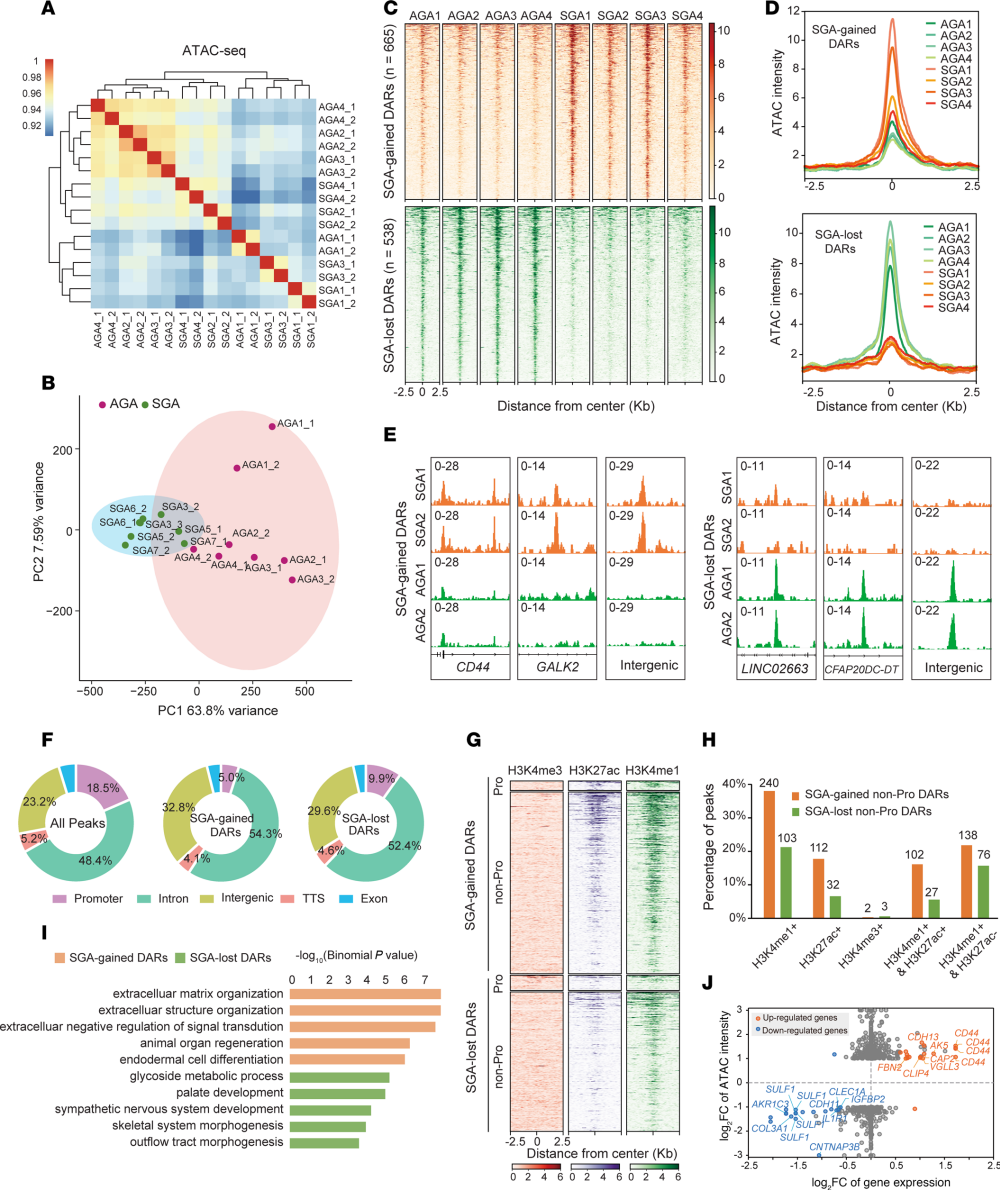
Genome-wide aberrant chromatin accessibility at enhancers in SGA-HUVECs. (**A**) Clustering of ATAC-Seq data by correlation of peak intensity between samples. (**B**) PCA of ATAC-Seq data based on peak intensity. (**C**) The ATAC-Seq signal enrichment around the peak center (±2.5 kb) of the 1,203 DARs. One technical replicate for each sample was shown. (**D**) Comparison of the ATAC-Seq signal density around the peak center (±2.5 kb) of the SGA-gained and SGA-lost DARs. (**E**) Selected examples of the SGA-gained and SGA-lost DARs. (**F**) Genomic annotation of DARs. The percentages of DARs in promoter, intron, exon, intergenic region, and transcription termination site are shown. (**G**) The enrichment of H3K4me3, H3K27ac, and H3K4me1 ChIP-Seq signals from HUVECs around the peak center (±2.5 kb) of the 1,203 DARs. DARs were classified as promoters and nonpromoters. (**H**) Percentage of peaks in nonpromoter DARs with different histone modifications. (**I**) GO analysis of top enriched biological processes in genes neighboring the SGA-gained or SGA-lost DARs. (**J**) Scatterplot showing the differential expression of the DARs nearest genes. *X* axis, log_2_ fold-changes of gene expression. *Y* axis, log_2_ fold-changes of ATAC-Seq peak intensity. DEGs were marked with red or blue color. The *y* axis scale value is limited to less than or equal to 3.

To gain insights into the epigenetic mechanisms associated with the dysregulated genes, ATAC-Seq analysis was performed to map genome-wide chromatin accessibility alterations with 2 technical replicates on samples from the same individuals used for RNA-Seq. All libraries were qualified with clear nucleosome phasing and enrichment of reads at transcription start sites as well as low mitochondrial DNA contamination ratio (average 10.50%) and high ratio of fraction of reads in peaks (average 27.00%) (Supplemental Figure 5, A and B). We defined overlapped peaks between technical replicates as high-confidence peaks in each sample and merged them in SGA and AGA groups. Overall, we detected 88,037 peaks in SGA and 107,647 peaks in AGA, which were further merged and resulted in a total of 111,564 peaks for the following analysis. Pearson’s correlation coefficients based on the pooled peaks and their intensity showed high correlations between samples within or between groups (>0.90), which did not separate SGA and AGA well. However, this was significantly improved by PCA, indicating the largest variance in ATAC-Seq datasets arose from SGA (Figure 4, A and B). To identify chromatin remodeling that contributes to the pathogenesis of SGA-HUVECs, we conducted differential analysis by DESeq2 and identified 1,203 differentially accessible regions (DARs) between SGA and AGA with a cutoff of |log_2_FC| ≥1 and *P* < 0.05. These DARs included 665 SGA-gained DARs and 538 SGA-lost DARs (Figure 4, C and D, and Supplemental Figure 5, C and D). Examples of DARs are shown in Figure 4E.

To gain insights into the function of DARs, we comprehensively analyzed their genomic features and nearby gene functions (Figure 4, F–I). Among the genome-wide 11,564 peaks, 18.50% overlapped with promoters, and the remaining nonpromoter peaks were primarily found in intergenic sites (23.20%) and introns (48.40%), which typically indicate active or poised enhancers (Figure 4F). Interestingly, compared with the overall distribution, the proportion of DARs within promoters decreased by 13.50% in SGA-gained DARs and 8.60% in SGA-lost DARs, while the proportion of DARs found in intergenic or intron regions increased, ranging from 4.00% to 9.60%, implying that enhancers were potential pathogenic factors. By integrating public ENCODE ChIP-Seq datasets featuring histone markers (H3K4me1, H3K4me3, and H3K27ac) obtained from HUVECs, we observed a remarkable enrichment of H3K4me1-marked enhancers and H3K27ac-marked active enhancers in DARs, particularly in the SGA-gained nonpromoter regions. In contrast, H3K4me3 signals were hardly observed as they typically occur in promoters (Figure 4G). Specifically, among the SGA-gained nonpromoter DARs, 38.00% were H3K4me1^+^, 17.70% were H3K27ac^+^, and 16.10% were H3K4me1^+^/H3K27ac^+^, whereas the proportions in SGA-lost nonpromoter DARs were 21.40%, 6.60%, and 5.60%, respectively (Figure 4H). As enhancers preferentially regulate nearby gene expression (37, 38), GO annotations of DAR-adjacent genes revealed significant enrichment of biological processes related to ECM in SGA-gained DARs (Figure 4I), which is consistent with the GO analysis in RNA-Seq.

Next, we analyzed the association between DEGs and DARs. Among the 223 upregulated DEGs in SGA, 19 were associated with SGA-gained DARs. Among the 176 downregulated DEGs in SGA, 20 were associated with SGA-lost DARs (Figure 4J). Significantly, *CD44*, as the top upregulated DAR-neighboring gene, was associated with 3 SGA-gained intronic DARs and 1 SGA-gained promoter DAR. *AKR1C3*, as the top downregulated DAR-neighboring gene, was associated with 3 SGA-lost intronic DARs. The highly downregulated gene *SULF1* was the nearest gene of 3 SGA-lost intronic DARs and 1 SGA-lost intergenic DAR. These findings implied that hyperactive angiogenic capacity of SGA-HUVECs is associated with aberrant chromatin accessibility at enhancers.

### Modulation of CD44 expression by its downstream active enhancers.

**Figure 5:**
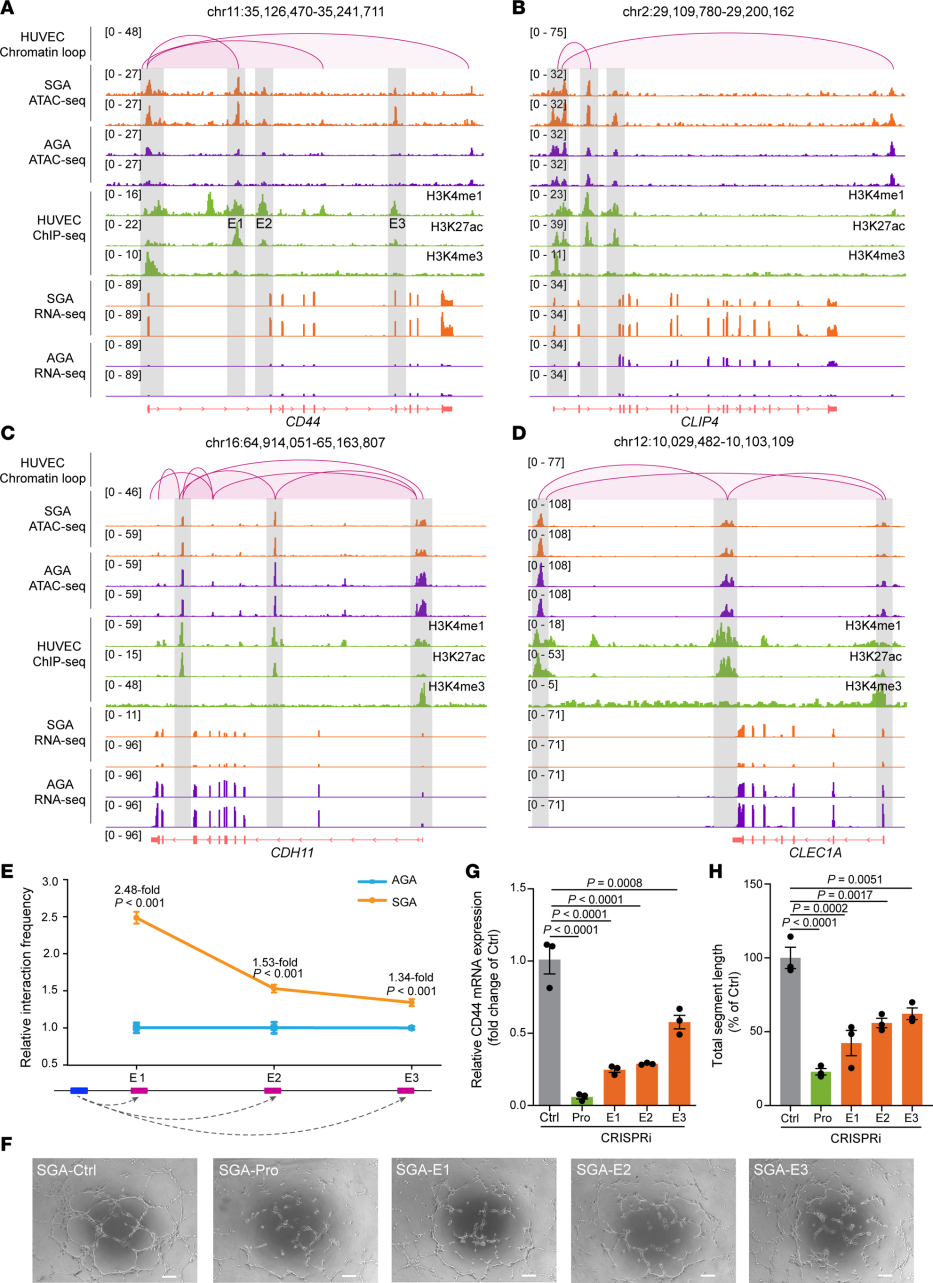
Characterization of enhancers regulating *CD44* expression. (**A**–**D**) Integrative Genomics Viewer track view of Hi-C, ATAC-Seq, ChIP-Seq (H3K4me1, H3K27ac, H3K4me3), and RNA-Seq normalized density of 4 representative examples of enhancer-promoter chromatin interaction. (**E**) 3C-qPCR reveals stronger promoter-enhancer interactions at *CD44* loci in SGA than AGA. Data are presented as mean ± SEM and analyzed by 2-tailed unpaired Student’s *t* test; *n* = 3. (**F**) Representative tube formation images of SGA-HUVECs with CRISPRi of *CD44* promoter (Pro) and its 3 downstream enhancers (E1, E2, E3); scale bar: 100 μm. (**G**) Lower *CD44* expression level in SGA-HUVECs with CRISPRi relative to vehicle; *n* = 3 per group. (**H**) Analysis of the total tube segment length for SGA-HUVECs with CRISPRi of *CD44* Pro and E1, E2, E3; *n* = 3 per group. Data in **G** and **H** are presented as mean ± SEM and were analyzed by 1-way ANOVA with Tukey’s multiple comparisons test.

Enhancers regulate distal gene expression through enhancer-promoter loops generated by chromatin folding, which can be detected by Hi-C (39). We extracted the DAR-associated interactions from the public ENCODE HUVEC Hi-C contact matrix (40) and identified 422 DAR-associated chromatin interactions. We further identified chromatin interactions between active enhancers (H3K4me1^+^/H3K27ac^+^) and active DEG promoters (H3K4me3^+^/H3K27ac^+^) associated with SGA-induced HUVEC dysfunction (Figure 5, A–D, and Supplemental Figure 6, A and B). For example, multiple pairwise Hi-C interactions were observed between *CDH11* promoter and 2 enhancers (Figure 5C). Specifically, located downstream of *CD44* promoter, 3 differentially accessible active enhancers (referred to as E1, E2, and E3) were identified (Figure 5A). Despite all 3 enhancers exhibiting strong H3K4me1 signals, E1 displayed the strongest H3K27ac and ATAC-Seq signals relative to E2 and E3, indicating that E1 is the most active enhancer in regulating *CD44* expression. Hi-C identified a chromatin loop only between E1 and *CD44* promoter, which was possibly because of the low depth of the public Hi-C datasets not being enough to report weak interactions. In addition, we performed chromosome conformation capture assay with quantitative PCR (3C-qPCR) to assess the physical interaction frequency between the *CD44* promoter and its enhancers. Compared with AGA-HUVECs, all interactions were significantly enhanced in SGA-HUVECs, with E1 exhibiting the highest interaction frequency with the promoter (Figure 5E), consistent with previous Hi-C data analysis.

To further validate the targeted regulatory relationship between the 3 enhancers and *CD44* expression, we employed CRISPRi to individually suppress the activity of E1, E2, and E3 by designing locus-specific guide RNA (gRNA) to direct a fused transcriptional repressor, dCas9-KRAB-T2a (41), to their respective targeted locus. As a positive control, we also utilized the same approach to perturb the promoter activity of *CD44*. RT-qPCR analysis of *CD44* mRNA levels verified the successful interference with *CD44* expression, revealing a 17.30-fold reduction after promoter CRISPRi and an average of 3.10-fold reduction after enhancer CRISPRi. The more pronounced effects observed with promoter interference suggest that the promoter is the most effective target locus in CRISPRi. For the 3 enhancers, E1 CRISPRi yielded the most significant perturbation results (4.10-fold reduction), followed by E2 (3.50-fold reduction), with E3 being less effective (1.80-fold reduction) (Figure 5G). Similar trends were observed in protein levels (Supplemental Figure 6C). This discrepancy may be associated with both sequentially decreased enhancer activity and their sequentially longer distances from *CD44* promoter, while we cannot rule out differences in gRNA efficiency. In line with the decreased *CD44* expression, a reduction of tube formation ability was observed in SGA-HUVECs (Figure 5, F and H, and Supplemental Figure 6, D and E). E1 showed a 2.30-fold reduction in total segment length, higher than the 1.80-fold reduction of E2 and the 1.60-fold reduction of E3, but all of them were less than the reduction in promoter interference. The extent of reduction entirely matched the trends observed in *CD44* expression changes, implying that *CD44* expression level is positively associated with angiogenesis. In summary, our results elucidate the significance of enhancers in SGA-HUVEC pathogenesis by integration of multiomics data and further validated the regulatory role of the 3 enhancers on *CD44* expression.

### Elevated AP-1 binding with CD44 promoter and enhancers drives the transactivation of CD44 in SGA-HUVECs.

**Figure 6:**
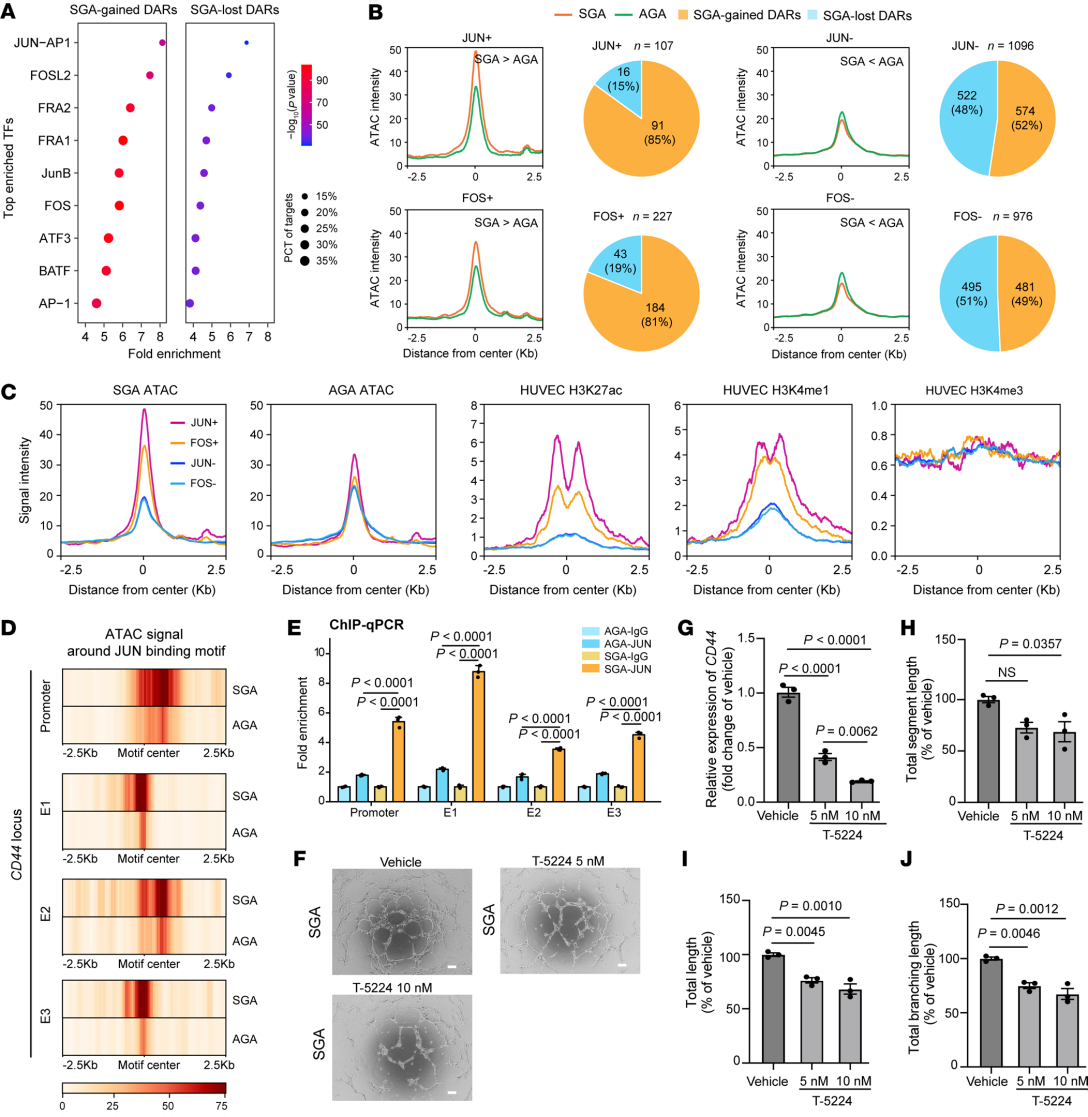
Regulation of *CD44* by AP-1. (**A**) TF motif enrichment in SGA-gained and SGA-lost DARs. The top 9 enriched TFs were shown. (**B**) Comparison of ATAC-Seq signal at DARs with or without JUN or FOS binding. The numbers of SGA-gained and SGA-lost DARs associated with FOS or JUN binding are shown in the pie chart. (**C**) Signal intensity of SGA and AGA ATAC-Seq and HUVEC histone markers (H3K27ac, H3K4me1, and H3K4me3) at JUN^+/–^ and FOS^+/–^ DARs. (**D**) ATAC-Seq signals around JUN binding motif at *CD44* Pro and downstream enhancers (E1, E2, E3). (**E**) SGA exhibits higher JUN binding activity at the *CD44* Pro and enhancers compared with AGA. (**F**) Representative tube formation images of SGA-HUVECs with T-5224 (AP-1 inhibitor) treatment; scale bar: 100 μm. (**G**) RT-qPCR shows reduced *CD44* expression level in SGA-HUVECs treated with T-5224; *n* = 3 per group. (**H**–**J**) Analysis of the total tube length, total tube branching length, and total segment length in SGA-HUVECs treated with T-5224; *n* = 3 per group. Data are presented as mean ± SEM and were analyzed by 1-way ANOVA with Tukey’s multiple comparisons test.

To investigate the TFs potentially bound to DARs, de novo motif analysis was conducted by HOMER (42). We identified 9 TFs that consistently ranked among the top enriched TFs in both SGA-gained and SGA-lost DARs (Supplemental Table 4). Remarkably, the majority of them are associated with the AP-1 regulatory complex, including known AP-1 subunits FOS, JUN, JUNB, FRA1, FRA2, and ATF3 (Figure 6A). AP-1 serves as a crucial TF complex in modulating the expression of essential genes for endothelial cell function and blood vessel formation during angiogenesis (43, 44). FOS and JUN are the most important subunits of AP-1. By integration of FOS and JUN ChIP-Seq datasets for HUVECs (45, 46), we found 18.87% (227 DARs) of DARs were bound by FOS, 8.89% (107 DARs) of DARs were bound by JUN, and 6.32% (76 DARs) of DARs were bound by both. SGA had a higher ATAC signal than AGA at JUN^+^ or FOS^+^ DARs but showed less ATAC signal at JUN^–^ or FOS^–^ DARs (Figure 6B and Supplemental Figure 7, A and B). Consistently, over 80% of DARs at JUN^+^ or FOS^+^ loci were SGA-gained DARs, while the proportion dropped to around 50.00% at JUN^–^ or FOS^–^ loci. Intriguingly, JUN^+^ DARs have higher ATAC signal than FOS^+^ DARs within SGA or AGA (Figure 6C). We further evaluated if the histone marker signal intensity was associated with FOS or JUN binding. In HUVECs, JUN^+^ DARs exhibited greater H3K27ac and H3K4me1 signal than FOS^+^ DARs (Figure 6C and Supplemental Figure 7B), suggesting a more active status at the JUN-bound locus. These observations suggest that JUN may preferentially bind to more accessible and active chromatin regions or that JUN binding could lead to a more open and active chromatin state.

We identified 242 DEGs with FOS or JUN binding at their promoters or nearby DARs, including *CD44* and *CDH11* (Supplemental Figure 7C). These genes were enriched in biological processes including response to growth factor, cell-substrate adhesion, and cell migration (data not shown), indicating AP-1 is the main TF regulating SGA-HUVECs’ dysfunction. We identified the JUN binding motif at both the *CD44* promoter and its 3 enhancers, with increased ATAC signal around the motifs in SGA compared with AGA (Figure 6D). We conducted JUN ChIP-qPCR in SGA and AGA cells to validate JUN binding activity on the *CD44* promoter and enhancers. As shown in Figure 6E, JUN exhibited significantly stronger enrichment on the *CD44* promoter and its 3 associated enhancers (E1, E2, E3) in SGA cells compared with AGA cells. To validate the regulatory role of AP-1 in *CD44* expression and HUVEC function, we treated SGA-HUVECs with T-5224, a widely used small molecule inhibitor that specifically inhibits the DNA binding activity of AP-1 (47). Compared with the vehicle, both low-dose and high-dose T-5224 treatments significantly reduced *CD44* expression and weakened tube formation ability in SGA-HUVECs, and greater reduction effects were observed with the high-dose treatment (Figure 6, F–J). These results highlight AP-1 as the primary transcription factor influencing SGA-HUVECs’ functions through its binding to both the *CD44* promoter and associated enhancers.

## Discussion

**Figure 7:**
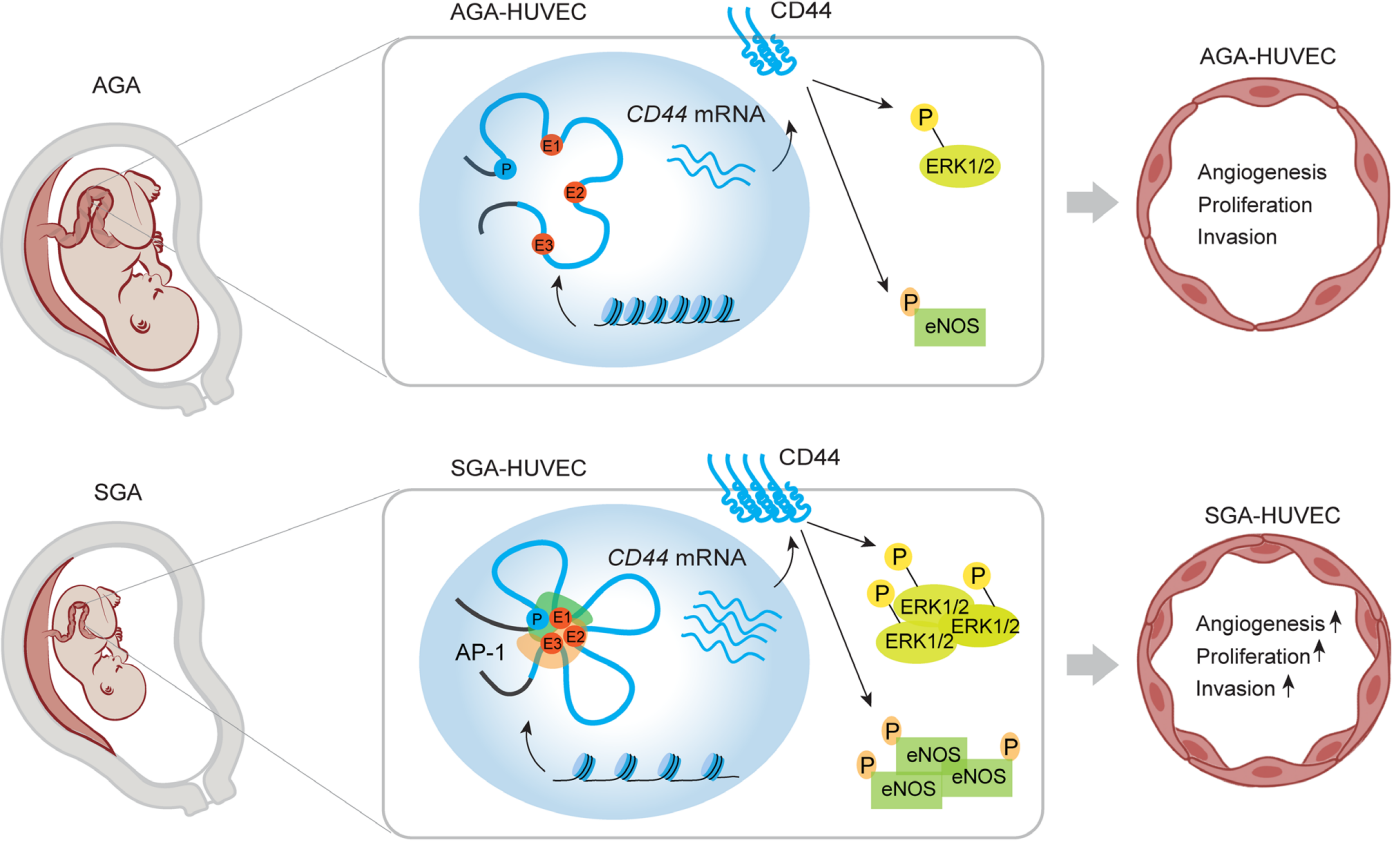
Proposed model for the epigenetic regulation mechanism of SGA-HUVECs’ dysfunction. *CD44* was identified as the primary pathogenic factor leading to the dysfunction of SGA-HUVECs by activating p-ERK1/2 and p-eNOS (Ser1177). The increased accessibility of 3 enhancers located downstream of the *CD44* promoter interacted with the *CD44* promoter through the formation of chromatin loops. Furthermore, AP-1 acts as a key transcription factor regulating elevated *CD44* expression by directly binding to both the *CD44* promoter and its associated enhancers, thereby reinforcing enhancer-promoter interaction loops.

HUVECs, forming the endothelial layer of the umbilical vein, are essential for maintaining fetal circulatory system development and homeostasis through their roles in angiogenesis and vascular tone regulation (48). Previous studies have reported impaired endothelium-dependent vasodilation and increased vascular stiffness in SGA fetuses (49, 50). This dysfunction aligns with the Developmental Origins of Health and Disease hypothesis, which links adverse intrauterine environments to epigenetic programming, increasing the risk of adult-onset diseases, such as pulmonary arterial hypertension and metabolic disorders (51, 52). Investigating HUVEC dysfunction in SGA is therefore crucial for identifying early interventions. In this study, we found that SGA-HUVECs exhibited enhanced angiogenic capacity, migration, and proliferation, suggesting significant endothelial remodeling. Comprehensive analyses identified *CD44* as a key pathogenic factor that activates p-ERK1/2 and p-eNOS (Ser1177). Its upregulation was attributed to increased chromatin accessibility at 3 enhancers, which interact with the *CD44* promoter through chromatin looping. AP-1, a pioneer transcription factor, was found to bind both the *CD44* promoter and enhancers, reinforcing these interactions and driving *CD44* expression (Figure 7). These findings reveal epigenetic regulation of *CD44* as a central mechanism in SGA-related endothelial dysfunction, offering potential therapeutic insights for fetal-origin, adult-onset diseases.

SGA infants, primarily resulting from reduced placental transfer of oxygen and nutrients, exhibit significant vascular adaptations (53). Consistent with previous reports (54), we observed smaller umbilical cord and vein diameters in SGA newborns compared with their AGA counterparts. This reduction in umbilical vein diameter likely reflects a physiological response to intrauterine oxygen and nutrient restriction driven by fetal, placental, or maternal factors (55). To sustain blood flow under these conditions, SGA-HUVECs demonstrated compensatory mechanisms, such as enhanced angiogenesis, proliferation, and migration. However, these adaptations often come at the cost of vascular integrity, contributing to endothelial dysfunction that is a key precursor to atherosclerosis, hypertension, and other cardiovascular diseases (56, 57). Basal levels of p-ERK1/2 were elevated in SGA-HUVECs compared with AGA-HUVECs, consistent with a previous study (11). Interestingly, we observed enhanced eNOS activation (Ser1177–p-eNOS/eNOS) in SGA-HUVECs. This contrasts with the findings of Casanello et al., where eNOS activity of SGA-HUVECs decreased following hypoxic stimulation (58). We speculate this discrepancy arises from the milder degree of hypoxia in the intrauterine environment, which may trigger a compensatory mechanism to mitigate the suboptimal conditions. Emerging evidence suggests that angiogenic switch is closely tied to metabolic shifts in endothelial cells (59), particularly through glycolysis, which provides the ATP supporting endothelial proliferation and migration in diseased vasculature. In SGA-HUVECs, we observed increased glucose consumption, potentially signifying a greater reliance on glycolysis. Further assessing glycolysis in SGA-HUVECs is indeed crucial to determine whether these cells undergo metabolic reprogramming. In our study, elevated ROS levels were also observed in SGA-HUVECs. Although moderate ROS levels can stimulate angiogenesis, excessive ROS induces oxidative stress, damaging cellular components and impairing vascular function (60). Thus, whereas increased glucose consumption may initially support angiogenesis in SGA-HUVECs, it could also predispose cells to oxidative damage, potentially compromising endothelial health over time. The maladaptive glucose metabolism and ROS generation may also provide a mechanistic link between early vascular adaptations in SGA individuals and their increased risk of adult-onset cardiovascular dysfunction. These findings suggest that the early vascular and metabolic adaptations in SGA infants could lay the foundation for the development of cardiovascular complications later in life.

CD44, a pro-angiogenic protein and a key regulator of endothelial migration, adhesion, and ECM remodeling (24), is critically involved in SGA-HUVEC dysfunction, which was supported by *CD44* gain- and loss-of-function interventions. Previous studies have linked *CD44* to diseases marked by impaired angiogenesis and insulin resistance, such as atherosclerosis, aging, and endothelial cell senescence (61–63). Knockdown of *CD44* in SGA-HUVECs disrupted angiogenesis-related pathways by downregulating pro-angiogenic genes (*LYVE1*, *SULF2*, *TFPI2*) and upregulating inflammatory and fibrotic genes (*VCAM1*, *IGFBP3*, *ADAMTS18*, *HAPLN1*), accompanied by a significant decrease in p-ERK1/2 and p-eNOS expression. These findings were consistent in AGA-HUVECS with low *CD44* expression and SGA-HUVECs with high *CD44* expression, suggesting *CD44*’s critical role in regulating endothelial cell function and vascular remodeling. These molecular alterations likely destabilize ECM dynamics, reduce growth factor bioavailability, and impair endothelial migration.

CD44 has been reported to be an aging-associated protein depending on its intercellular domain (61, 62). Studies have shown that *CD44* undergoes age-related upregulation and plays a crucial role in vascular endothelial cell senescence (61). *CD44* promotes endothelial cell senescence by modulating autophagy, regulating inflammation, and altering key cell signaling pathways. In animal models (both mice and rats), increased *CD44* expression has been observed in endothelial cells, emphasizing its role in vascular aging (61, 62). In SGA infants, elevated *CD44* expression in SGA-HUVECs may indicate an early onset of vascular aging in endothelial cells, potentially predisposing individuals to cardiovascular diseases over the long term. Notably, accelerated placental aging has been observed in SGA infants (64), and a clinical trial found that being born SGA is associated with early vascular aging in adolescents (65). These findings support the concept that early vascular changes in SGA infants may predispose them to cardiovascular issues later in life. While *CD44* has been implicated in both aging and endothelial cell senescence, it remains an open question whether its upregulation in SGA individuals follows an age-dependent pattern. This presents an exciting avenue for future research to explore the potential role of *CD44* in the vascular aging process in SGA infants.

We also provide compelling evidence for the role of epigenetic remodeling and chromatin reorganization in the endothelial dysfunction in SGA. Enhancer-promoter interactions are critical for precise gene expression regulation, particularly in response to environmental or developmental stimuli (66). We identified numerous DARs in SGA-HUVECs, with significant increases in chromatin accessibility at *CD44* enhancers associated with elevated *CD44* expression, which highlights how chromatin dynamics activate gene expression programs driving pathological angiogenesis. Functional studies using CRISPRi-mediated enhancer silencing confirmed their importance in angiogenesis, suggesting these enhancers as potential therapeutic targets for fine-tuning *CD44* expression. Importantly, this enhanced accessibility facilitates increased binding of AP-1, a pioneer transcription factor known to initiate chromatin remodeling and shape 3-dimensional enhancer-promoter interactions (67). In SGA-HUVECs, AP-1 binding mediates the strengthening of *CD44* promoter-enhancer interactions, suggesting its central role in reorganizing the chromatin landscape. This intricate interplay between chromatin accessibility, transcription factor activity, and 3-dimensional chromatin architecture revealed multilayered regulatory mechanisms that drive endothelial dysfunction in SGA. Interestingly, the interaction between CD44 intracellular domain and AP-1 promotes CD44 expression in breast cancer cells (68). Future studies should investigate whether this mechanism overlaps with ERK/eNOS-dependent signaling to amplify pathological responses.

Our study has several limitations. The relatively small sample size may limit the generalizability of our conclusions. Although the observed effects reached statistical significance, a larger cohort would be required to confirm the reproducibility of *CD44*’s role in SGA-associated endothelial dysfunction. Additionally, HUVECs may not fully represent adult vascular cells, as endothelial cell characteristics vary by region and age. To confirm the role of *CD44* in fetal programming and its contribution to adult-onset diseases, in vivo models or primary endothelial cells from adult tissues are necessary. Furthermore, while our data suggest CD44’s interaction with ERK/eNOS, the precise molecular mechanisms remain incompletely resolved. Future studies should also explore how CD44, as a surface protein, coordinates with other proteins like LYVE1 to regulate endothelial function.

## Methods

### Sex as a biological variable.

The number of female and male samples used in our study was balanced. The sex information is shown in Supplemental Table 1. Sex was not considered as a biological variable. These findings are expected to apply to both sexes.

### HUVECs’ isolation and culture.

The umbilical cord was obtained immediately after delivery and transferred to a precooled, sterile phosphate-buffered saline (PBS) containing heparin sodium and antibiotics. Before cell isolation, approximately 1 cm of the umbilical cord tissue was cut off perpendicular to the long axis for immunohistochemistry. Then HUVECs were isolated as described previously (12). These endothelial cells were cultured in endothelial cell medium, with 20% fetal bovine serum (FBS), endothelial cell growth supplement, and antibiotic solution (1001, Sciencell), at 37°C with 5% CO_2_. All HUVECs used in the current study were obtained within passage 6. For drug experiments, cells were cultured in media with 5 nmol/L and 10 nmol/L T-5224, respectively (HY-12270, MedChemExpress), for 24 hours to inhibit AP-1.

### Immunofluorescence.

HUVECs were seeded on 6-well plates and cultured until 70%–80% confluence. Cells were fixed with 4% paraformaldehyde for 15 minutes and permeabilized with 0.1% Triton X-100 for 10 minutes. Then 10% goat serum was used for blocking at room temperature for 1 hour. Cells were incubated with the CD31 antibody (ab9498, 1 μg/mL, Abcam) and the vWF antibody (ab154193, 1:500, Abcam) at 4°C overnight. IgG was used as negative control at the same time. The next day, cells were incubated by secondary antibody (A0428 and A0468, Beyotime), and cell nuclei were stained with DAPI. Fluorescent images were captured by a fluorescent microscope (ZEISS Observer Z1).

### CCK8 assay.

CCK8 assay (A311, Vazyme) was performed to examine the proliferation of HUVECs as per manufacturer’s recommendations. Briefly, HUVECs were seeded at a density of 2,000 cells per well in a 96-well plate and incubated at 37°C with 5% CO_2_ in a humidified incubator. At various time points (0 hours, 24 hours, 48 hours, and 72 hours), the CCK8 solution was added to sample wells and incubated for 3 hours in the incubator. The absorbance at 450 nm was measured using a microplate reader (Synergy Neo2, BioTek).

### EdU assay.

Proliferative ability was assessed following the instructions of the EdU kit (C10310-1, Ribobio). Briefly, HUVECs were seeded at a density of 5,000 cells per well in 96-well microplates and then incubated with EdU for 2 hours. Subsequently, cells were fixed, washed, and incubated in glycine, followed by permeabilization using 0.5% Triton X-100 for 10 minutes. Apollo staining buffer was applied for 30 minutes at room temperature in the dark. Finally, Hoechst 33342 was utilized to stain the cell nuclei. The fluorescence microscope used for imaging was ZEISS Observer Z1.

### Capillary tube formation assay.

Tube formation assay was performed in precooled, 96-well plates coated with Matrigel Basement Membrane Matrix (BD356234, Corning). A total of 5,000 HUVECs per well were seeded into the plates and incubated at 37°C with 5% CO_2_ for 6 hours. Photographs were obtained by microscope (ZEISS Observer Z1), and image analysis was performed by ImageJ software (NIH).

### Migration assay.

To assess the migration ability of HUVECs, we used the Transwell chamber migration assay (3422, Corning). A total of 1 × 10^5^ cells were resuspended in serum-free medium and added to the upper chamber, while the lower chamber was supplemented with 10% FBS. Following 24 hours of incubation, noninvaded cells were carefully removed from the upper surface of the filter. The invaded cells were subsequently fixed and stained. To quantify cell migration, the average number of cells in 5 visual fields was determined.

### Scratch wound healing assay.

The scratch assay was performed to detect the cell migration rate. Primary HUVECs were seeded on a 6-well plate until the confluence reached 100% and then were scratched by a sterile 200 μL pipette tip. Cells were washed gently with PBS and then cultured in ECM without FBS. Images of the wounded area were taken at 0 and 12 hours. At least 5 random, nonoverlapping images per experiment were analyzed and quantitated using ImageJ software.

### Glucose consumption assay.

The glucose content in the conditioned medium was measured according to the instructions of the Glucose Assay Kit (60408ES, Yeasen). Briefly, HUVECs (2 × 10^5^ cells/mL) were cultured in 12-well plates, and the medium was replaced with serum-free ECM when cells reached 90% confluence. After 24 hours of incubation, we added 250 μL working solution to 2.5 μL samples and incubated for 10 minutes at 37°C. The absorbance value was read at 505 nm by a multiscan spectrum (Synergy Neo2). The concentration of glucose was determined as the concentration of glucose in serum-free ECM minus the concentration of glucose in the supernatant after 24 hours.

### ROS assay.

ROS levels were quantified using the ROS Assay Kit (S0033S, Beyotime). Briefly, HUVECs were seeded at a density of 5,000 cells per well in 96-well microplates and incubated at 37°C with 5% CO_2_. After 24 hours of incubation, cells were treated with 10 μM DCFH-DA at 37°C for 20 minutes. Subsequently, cells were washed 3 times with PBS. Fluorescence intensity at 488 nm excitation and 525 nm emission was measured using a fluorescence plate reader (Synergy Neo2).

### RNA-Seq library construction and data processing.

Total RNA was extracted from frozen samples using TRIzol reagent (Invitrogen, Thermo Fisher Scientific), followed by mRNA enrichment using poly-T oligos coated on magnetic beads. The enriched mRNA was subjected to stranded RNA-Seq library construction as described previously (69). The final libraries were subjected to paired-end 150 bp sequencing on the Illumina NovaSeq 6000. Raw reads were trimmed using trim_galore (v0.6.10) and mapped to the hg38 genome using HISAT2 (v2.2.1). The transcripts were quantified with htseq-count (v2.0.4). Genes with maximum FPKM values below 1.0 in all samples were defined as unexpressed and excluded from downstream analysis. Sample clustering based on the FPKM matrix was performed using cor function with pearson method in R program v4.3.1, then visualized using R package pheatmap (v1.0.12) and ggplot2 (v3.4.4). Differential expression was analyzed using Cuffdiff (70). Genes with adjusted *P* value < 0.05 and fold-change (FPKM + 1) ≥ 1.5 were assigned as DEGs. GO analysis of DEGs was performed with ShinyGO with default parameters (71). The PPI network was set up through STRING (72) and visualized using Cytoscape (v3.7.1).

### RT-qPCR.

Total mRNA was extracted from HUVECs according to the instructions of the Total RNA Kit (Axygen). Subsequently, the RNA was transcribed into cDNA using the reverse transcriptase kit (Takara). The synthesized cDNA was analyzed using the StepOnePlus Real-Time PCR system, following the SYBR-Green protocol (Takara). The PCR conditions were as follows: 95°C for 30 seconds, followed by 40 cycles of 5 seconds at 95°C and 30 seconds at 60°C. The relative expression of target genes to GAPDH was quantified using the 2^–ΔΔCt^ method. Primers are listed in Supplemental Table 5.

### IHC.

IHC was conducted on umbilical cord tissues following previously established protocols (11). In brief, the paraffin-embedded tissues were sectioned at a thickness of 4–5 μm. The sections were then subjected to deparaffinization using xylene and rehydration through a series of graded ethanol concentrations until reaching distilled water, followed by antigen retrieval. Subsequently, the samples were incubated in methanol containing 3% hydrogen peroxide for 25 minutes to quench endogenous peroxidase activity and washed with PBS. Next, the sections were incubated with the primary antibody anti-CD44 (A19020, 1:200, Abclonal). The quantitative analysis of CD44-positive cells was done by counting manually. Each group had 4 individuals, and at least 6 nonoverlapping fields of view from each section were counted.

### Western blot.

HUVECs were lysed using RIPA buffer (P0013B, Beyotime) supplemented with protease and phosphatase inhibitor cocktails. The protein lysates were separated on a 10% sodium dodecylsulfate (SDS) polyacrylamide gel electrophoresis and subsequently transferred to polyvinylidene difluoride membranes (Merck Millipore). The following primary antibodies were used: CD44 (60224, 1:5,000, Proteintech), p-ERK1/2 (4370, 1:1,000, Cell Signaling Technology [CST]), t-ERK1/2 (4695, 1:1,000, CST), p-eNOS (A20985, 1:1,000, Abclonal), t-eNOS (AP1404,1:1,000, Abclonal), β-actin (8457S, 1:1,000, CST), and GAPDH (60004-1-Ig, 1:50,000, Proteintech).

### 3C-qPCR.

The 3C-qPCR assay was performed as previously described (73), with minor modifications. In brief, 2 × 10^6^ formaldehyde-cross-linked SGA or AGA cells were lysed with 0.3% SDS and then incubated in a digestion reaction containing 100 U of DpnII (R0543S, New England Biolabs, NEB) at 37°C overnight. After heat inactivation at 62°C for 10 minutes, the nuclei were pelleted and subjected to a ligation reaction with 2,000 U of T4 DNA ligase (M0202L, NEB) at 16°C overnight. The ligation products were de-cross-linked and purified using the ChIP DNA Clean & Concentrator Kit (D5205, Zymo). The purified DNA was quantified with Qubit-4 and diluted to approximately 180 ng/μL for qPCR. Primers used are listed in Supplemental Table 5.

### ChIP-qPCR.

A total of 2 × 10^6^ formaldehyde-cross-linked SGA or AGA cells were lysed and sheared using a Sonics Vibra Cell (VC505) at 25% amplitude for 10 cycles (30 seconds on, 30 seconds off). One percent of the chromatin was reserved as an input control. Chromatin was incubated with antibodies (anti-JUN: 24909-1-AP, Proteintech; IgG: RGAR001, Proteintech) and a mixture of Dynabeads Protein A for Immunoprecipitation (10002D, Thermo Fisher Scientific) and Protein G (10004D, Thermo Fisher Scientific) at 4°C for 2 hours. The precleared chromatin was then incubated with the corresponding antibodies at 4°C overnight. Antibody-chromatin complexes were washed and eluted. The purified immunoprecipitated DNA and input DNA were analyzed by qPCR using primers targeting AP-1 binding sites in the *CD44* promoter or related enhancers, as listed in Supplemental Table 5.

### ATAC-Seq library construction and data processing.

The ATAC-Seq libraries were constructed with TruePrep DNA library prep kit V2 for Illumina (TD501, Vazyme) as previously described with minor modifications (74). Briefly, 25,000 cells were lysed with 50 μL cold lysis buffer (10 mM Tris-HCl pH 7.4, 10 mM NaCl, 10 mM MgCl_2_, 0.1% [v/v] NP-40, 0.1% [v/v] Tween 20, 0.01% [v/v] Digitonin) on ice for 3 minutes and then tagmented with Tn5 by incubating at 37°C for 30 minutes on thermomixer (GET3XG, BIO-GENER). The reaction was stopped by addition of 0.5% SDS incubating at 65°C for 10 minutes. Tagmented DNA was purified by ChIP DNA clean & concentrator kit (D5205, Zymo) and amplified by 12 cycles of PCR. Libraries were cleaned up and sequenced on Illumina NovaSeq 6000 platform.

Raw reads were trimmed using trim_galore (v0.6.10) (75) and aligned to the hg38 genome using Bowtie2 (v2.5.1). Reads with low mapping quality (MapQ < 30), PCR duplicates, and mitochondrial DNA were removed with picard (v2.27.4) and samtools (v1.18). The peaks were called using MACS2 (v2.2.9.1) (76) with the parameter --shift -100 --extsize 200 --qvalue 0.01 --nomodel -B --SPMR --keep-dup all, and then we merged peaks across all samples using BEDTools merged with default parameters to get a peak reference (v2.29.1). To quantify the activity of each peak and conduct differential analysis, read depths in a peak region were calculated using the SAMtools bedcov utility and normalized with respect to the FPKM value using the DESeq2 R package (v1.40.2); the normalized value was defined as the peak activity. Clustering of the samples based on peak activity was performed using cor function with pearson method in R program v4.3.1, then visualized using R package pheatmap (v1.0.12) and ggplot2 (v3.4.4). We used the DESeq2 program to identify peaks that showed differential accessibility between the 2 groups. Regions with *P* value < 0.05 and fold-change ≥ 2 were assigned as DARs.

### Annotation and functional analysis of DARs.

The DAR annotation and motif enrichment were conducted by HOMER (v4.4) (42). The database of vertebrate known TF motifs was inquired with default parameters. GO enrichment analysis of DARs was performed using the Genomic Regions Enrichment of Annotations Tool with minimum term annotation count set as 10 (77). The BAM alignment files were converted to bigWig format and normalized by scaling factor (--scale- Factor) with the deepTools (v3.5.4) bamCoverage function. The bigWig files and DARs coordinates were used as input for the computeMatrix function of deepTools. This matrix was used as input in plotHeatmap and plotProfile function for visualization. To identify DARs with histone modifications and TF binding, the DARs were overlapped with downloaded peaks with at least 1 bp overlap. To identify DAR-associated chromatin interactions, interactions with observed frequency ≥ 5 and having at least 1 bp overlap with DARs at left or right anchors were counted.

### Knockdown of CD44 in SGA-HUVECs.

*CD44* knockdown was accomplished through the utilization of Si-*CD44*. HUVECs underwent transient transfection with siRNA transfection reagent (40806ES, Yeasen) in adherence to the manufacturer’s instructions. Sequences are listed in Supplemental Table 5. Cells transfected with negative siRNA served as the experimental control. The assessment of knockdown efficiency was conducted using RT-qPCR and Western blot analysis at 48 hours and 72 hours after transfection, respectively.

### CRISPRi and CD44 overexpression.

DNA sequences encoding small guide RNA (sgRNA) were designed using the CHOPCHOP online tool (Supplemental Table 5 for oligo sequences). For CRISPRi, the sequences were inserted into pLV-hU6-sgRNA-hUbC-dCas9-KRAB-T2a-Puro (71236, Addgene). For overexpression, the *CD44* gene (NM_000610.4) was inserted into the expression vector pCDH-CMV-MCS-EF1-PURO (Addgene).

### Production of lentiviral medium.

Active lentiviral medium was performed with third-generation lentiviral transfer plasmids (1,000 ng each) mixed with 1,000 ng of a packaging DNA premix using psPAX2 (12260, Addgene) and pMD2.G (12259, Addgene) in a 3:1 ratio, which were transfected into Lenti-X 293T cells (Procell) using Hieff Trans Liposomal Transfection Reagent (40802ES, Yeasen) according to the manufacturer’s instructions in a 6-well plate. The transfection mixture was added to Lenti-X 293T cells cultured in DMEM containing 10% FBS. Lentivirus-containing conditioned medium was collected after 48 hours, centrifuged at 1,000*g* for 5 minutes, filtered at 0.45 μm, and stored at –80°C.

### Infection with lentiviral medium.

HUVECs (1.5 × 10^5^ cells/mL) were cultured in 12-well plates, and the medium was replaced with ECM complete medium containing 500 μL virus supernatant and 8 μg/mL polybrene (40802ES, Yeasen) after 70%–80% confluence. Transfected cells were selected with puromycin (1 μg/mL) starting 48 hours postinfection. Then, the alive cells were collected to verify the efficiency of CRISPRi by RT-qPCR and Western blot analysis.

### Statistics.

Unless otherwise stated, the statistical analyses were performed in the R program (v4.3.1). GraphPad Prism 9.0 and SPSS Statistical package (version 27; SPSS) were used for statistical analysis. Two-tailed unpaired Student’s *t* tests were used for comparison between 2 groups. One-way ANOVA and 2-way ANOVA were used for multiple comparisons. Details of statistical tests used are specified in figure legends. *P* < 0.05 was considered significant. All results are presented as mean ± SEM.

### Study approval.

All samples used in this study was approved by the Clinical Research Ethics Committee of the First Affiliated Hospital, Zhejiang University School of Medicine (IIT20220268B-R1), conforming to the principles outlined in the Declaration of Helsinki concerning the use of human tissue samples. Informed consent documents were signed by parents prior to delivery. Fetal growth-restricted and normal umbilical cord tissue samples were from the First Affiliated Hospital, Zhejiang University School of Medicine. SGA was defined as birth weight below the 10th percentile of the identical gestational age and sex according to the growth chart of Chinese newborns (23). Control was described as AGA, with birth weight range from the 10th to 90th percentile, without intrauterine infection or any other medical or obstetrical complication, whose mother was normotensive, nonsmoking, and non-alcohol- or drug-consuming.

### Data availability.

The raw data for ATAC-Seq and RNA-Seq reported in this paper have been deposited in the Genome Sequence Archive (78) in National Genomics Data Center (79), China National Center for Bioinformation/Beijing Institute of Genomics, Chinese Academy of Sciences (GSA-Human: HRA006615), and are publicly accessible at https://ngdc.cncb.ac.cn/gsa-human Processed ChIP-Seq and Hi-C data of the HUVEC line were downloaded from the ENCODE website (https://www.encodeproject.org/), including H3K4me3 (ENCFF161GMO and ENCFF992YLK), H3K27ac (ENCFF955PAU and ENCFF077LGZ), H3K4me1 (ENCFF254KUQ and ENCFF213BAF), FOS (ENCFF972ZIV and ENCFF301XXM), JUN (ENCFF624TOT and ENCFF672FUO), and Hi-C data (contact domains and loops: ENCFF904UGB and ENCFF174NVV). Raw data can be found in the Supporting Data Values file.

## Author contributions

LY, ZZ, and SC conceptualized the study under supervision of YZ, LG, and CW. LY, ZZ, and SC contributed equally to this work. LY designed the study, drafted the manuscript, and coordinated the project. ZZ analyzed the data and revised the manuscript. SC performed library construction and a portion of cell experiments. The authorship order reflects their respective contributions. LY, JM, XF, FL, JZ, XC, YH, and YW collected the clinical samples. LY, SC, and XF performed all experiments. ZZ, LY, XF, YZ, and LG performed the data analysis. LY, ZZ, and SC wrote the original draft. YZ, LG, CW, LD, and BW contributed to the review and editing of the manuscript. All authors reviewed the manuscript.

## Supplementary Material

Supplemental data

Unedited blot and gel images

Supporting data values

## Version 1 04/22/2025

Electronic publication
