# Supplementary material for: Reshaping the chromatin landscape in HUVECs from small-for-gestational-age newborns
Source: JCI Insight. 2025 Apr 22;10(8):e186812. doi: 10.1172/jci.insight.186812 (PMC12038915; doi:10.1172/jci.insight.186812)
Supplement: Supplemental data [file jciinsight-10-186812-s008.pdf]

Supplemental materials

for

Reshaping the chromatin landscape in HUVECs from small for gestational age newborns

Supplemental Figures

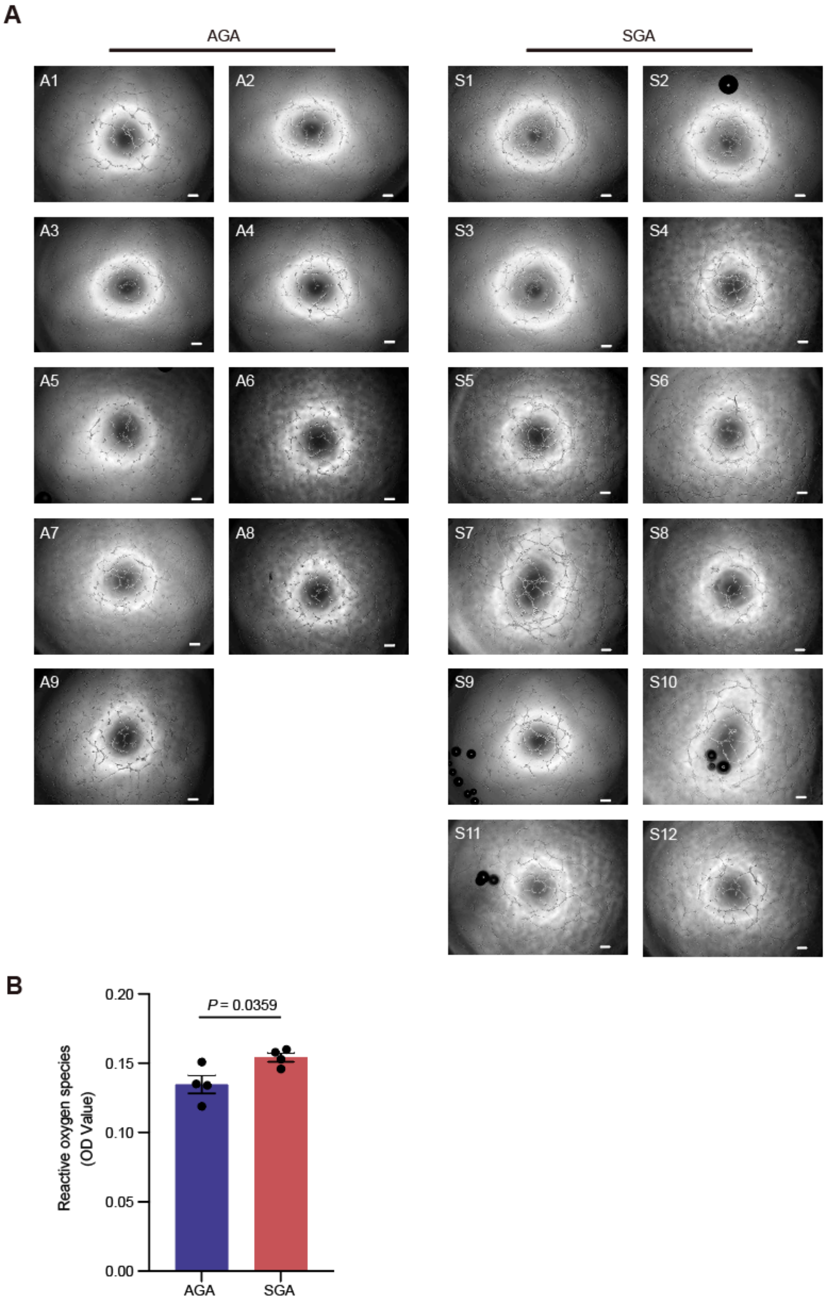

Supplemental Figure 1. (**A**) Representative images of tube formation assay for all HUVEC samples, scale bar: 200 μm**.** (**B**) Reactive oxygen species levels in both AGA and SGA HUVECs, n = 4 per group. Data presented as mean ± SEM and was analyzed by two-tailed unpaired Student's t test.

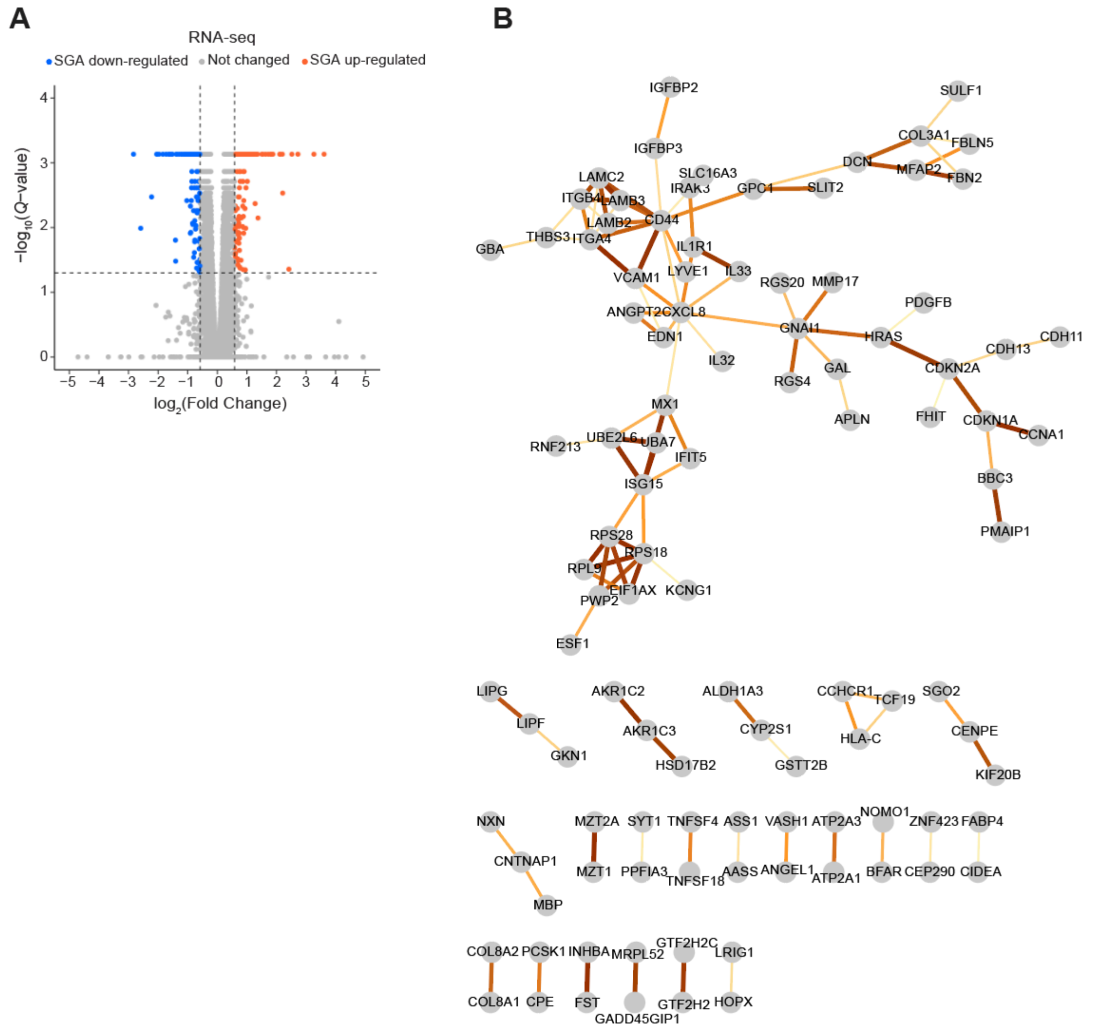

Supplemental Figure 2. Volcano plots and PPI analysis of RNA-seq data. (A) Volcano plots depict the differential gene expression between SGA and AGA. DEGs with Fold Change (FPKM+1) ≥ 1 and $Q < 0.05$ were colored with red or blue. **(B)** The full PPI network derived from all DEGs. Only interactions with confidence score ≥ 0.7 were used for analysis.

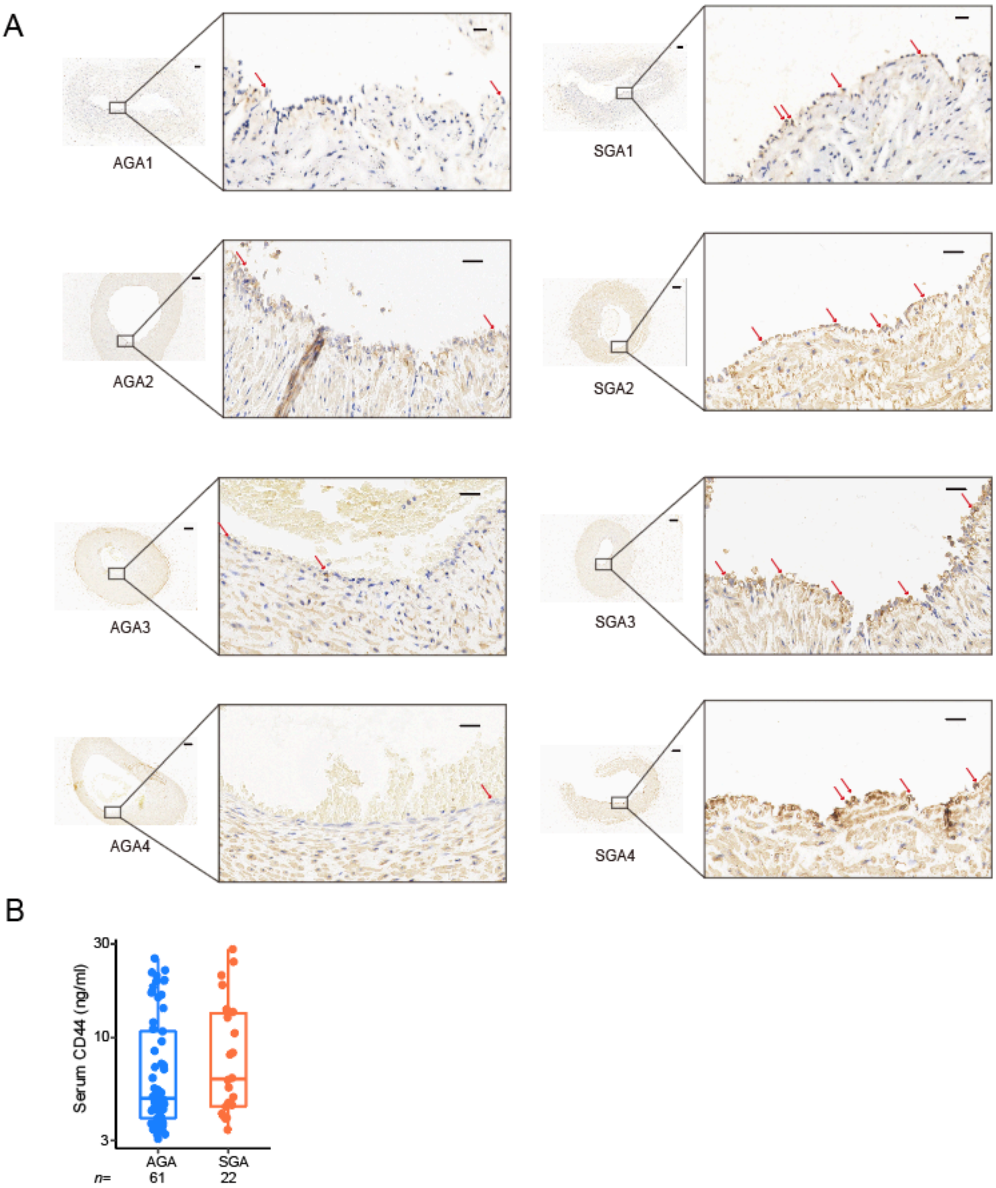

Supplemental Figure 3. Expression of CD44 in SGA relative to AGA. (A) Representative immunohistochemical images of paraffin-embedded umbilical cord samples from AGA and SGA using CD44 antibody. Red arrows indicate CD44 positive HUVECs. Scale bars: 200 μm (left) and 25 μm (right). **(B)** Analysis of CD44 protein levels in serum of SGA and AGA individuals.

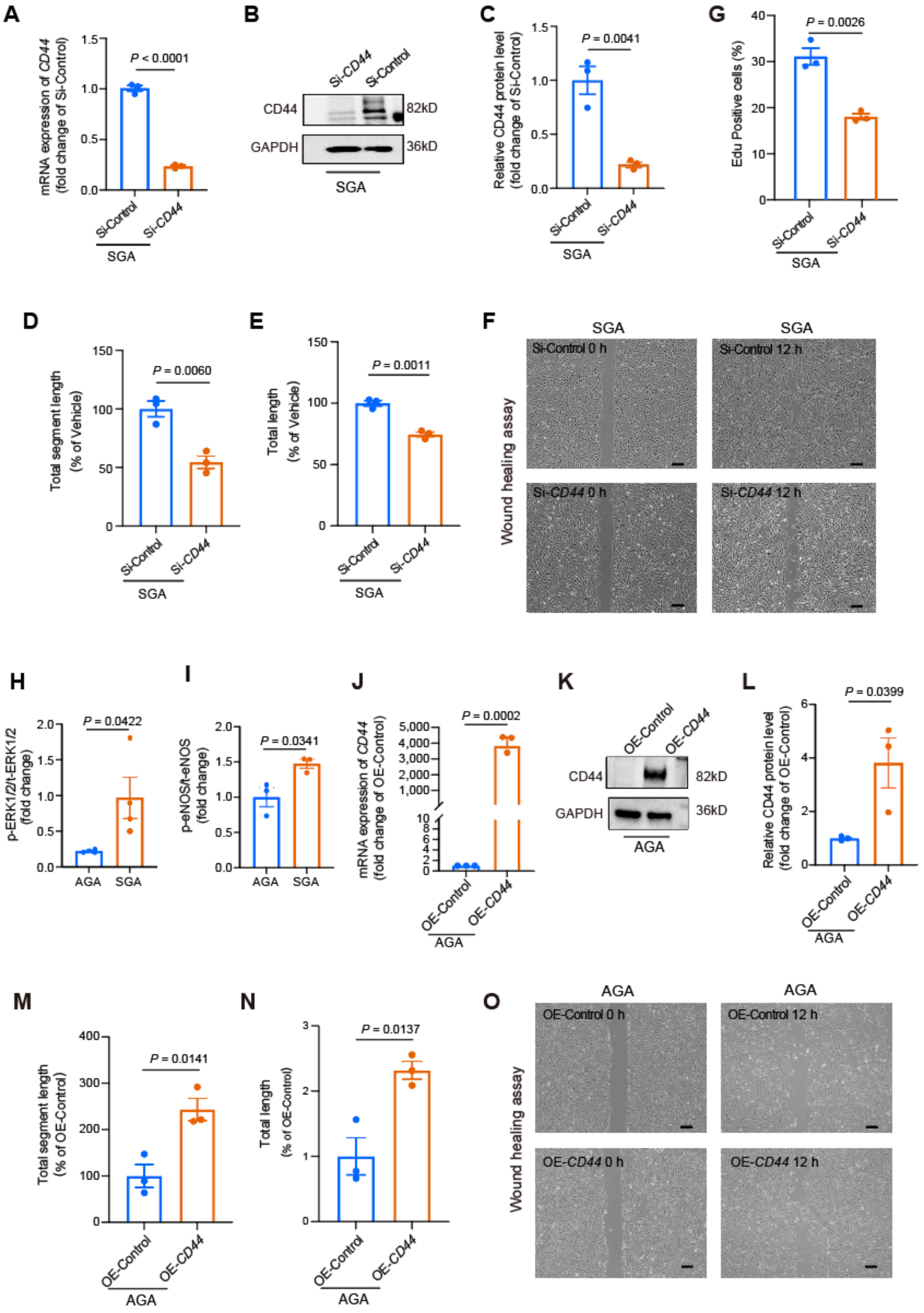

Supplemental Figure 4. Expression of *CD44* after knockdown and overexpression intervention and relative functional changes in SGA and AGA-HUVECs. (**A**) RT-qPCR shows reduced *CD44* expression level

in SGA-HUVECs with *CD44* knockdown, *n* = 3 per group. (**B** and **C**) Western blot shows the lower CD44 protein levels in SGA-HUVECs with *CD44* knockdown, *n* = 3 per group. (**D** and **E**) Analysis of the total segment length and total tube length for SGA-HUVECs with *CD44* knockdown, *n* = 3 per group. (**F**) Representative images of scratches at 0 h and 12 h in SGA-HUVECs with *CD44* knockdown, scale bar: 200 µm. (**G**) Analysis of EdU assay result of SGA-HUVECs with *CD44* knockdown, *n* = 3 per group. (**H** and **I**) Analysis of p-ERK/t-ERK detected by western blot, *n* = 3-4 per group. (**J**) RT-qPCR shows elevated *CD44* expression level in AGA-HUVECs with *CD44* overexpression, *n* = 3 per group. (**K** and **L**) Western blot shows the increased CD44 protein levels in AGA-HUVECs with *CD44* overexpression, *n* = 3 per group. (**M** and **N**) Analysis of the total segment length and total tube length for AGA-HUVECs with *CD44* overexpression, *n* = 3 per group. (**O)** Representative images of scratches at 0 h and 12 h in AGA-HUVECs with *CD44* overexpression, scale bar: 200 µm. Data are presented as mean ± SEM and were analyzed by two-tailed unpaired Student's *t* test.

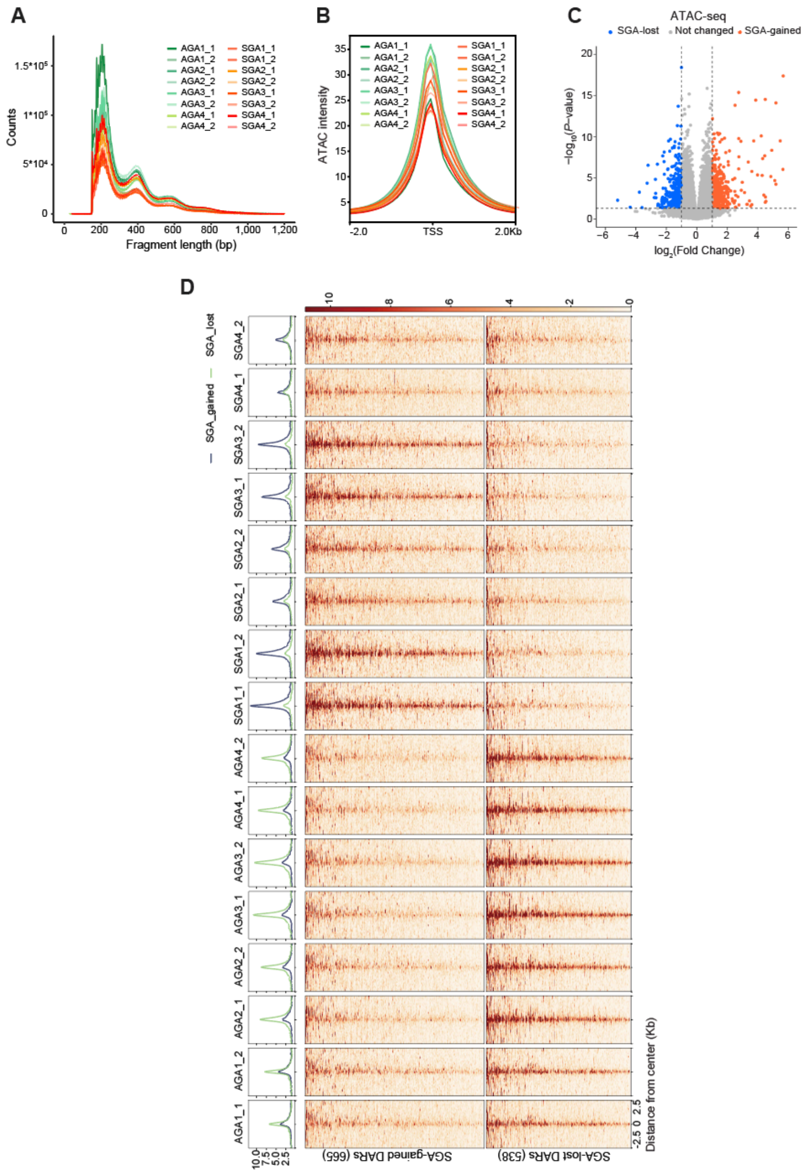

Supplemental Figure 5. Quality control and analysis of ATAC-seq data. **(A)** Distribution of insert fragment sizes in ATAC-seq libraries. **(B)** Enrichment of ATAC-seq signal around the TSS (± 2 Kb). **(C)** Volcano plots depict the differential accessible regions between SGA and AGA. DARs with Fold Change ≥ 2 and $P < 0.05$ were colored with red or blue. **(D)** ATAC-seq signal enrichment around the peak center (± 2.5 Kb) of DARs in all datasets. All samples were shown with two technical replicates.

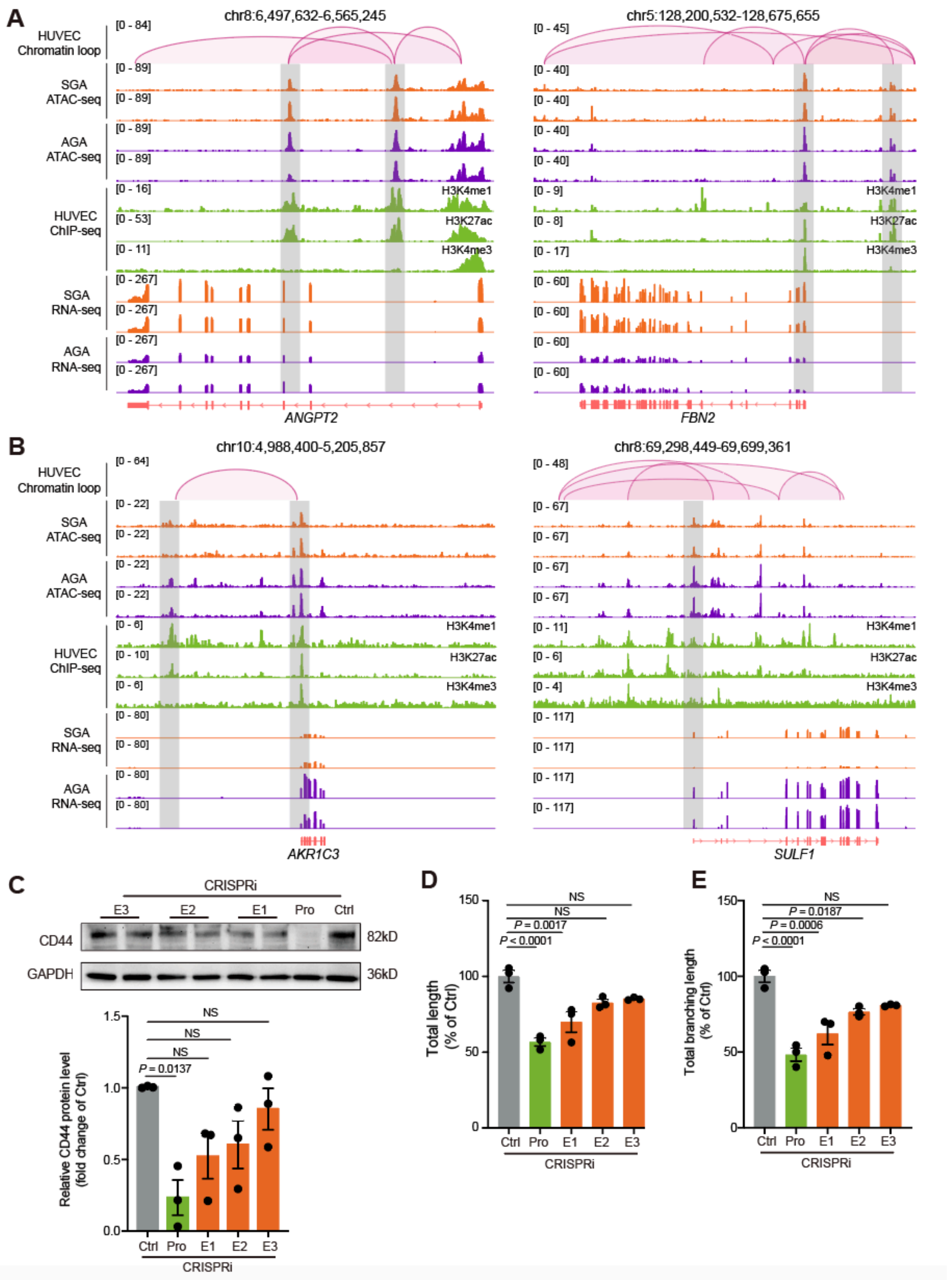

Supplemental Figure 6. Characterization of enhancers regulating *CD44* expression. (A-B) IGV track view of Hi-C, ATAC-seq, ChIP-seq (H3K4me1, H3K27ac, H3K4me3), RNA-seq normalized density of four representative examples of enhancer-promoter chromatin interaction. **(C)**

Western blot shows the lower CD44 protein levels in SGA-HUVECs with CRISPRi of *CD44* promoter (Pro) and its three downstream enhancers (E1, E2, E3) relative to vehicle, n = 3 per group. **(D)** Analysis of the total tube length and total tube branching length for SGA-HUVECs with CRISPRi of *CD44* promoter (Pro) and its three downstream enhancers (E1, E2, E3), n = 3 per group. Data presented as mean ± SEM and were analyzed by one-way ANOVA with Tukey's multiple comparisons test. NS, nonsignificant.

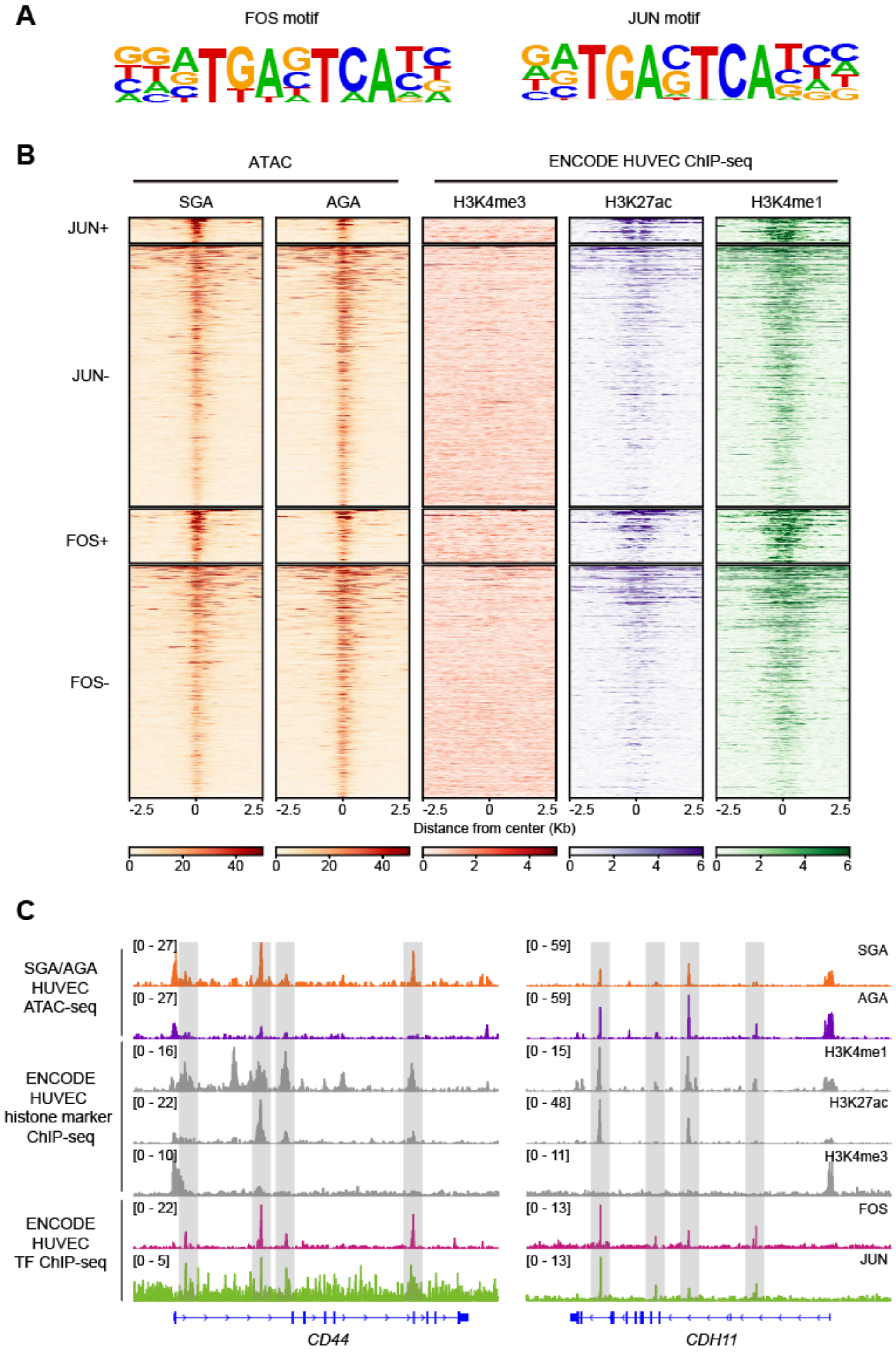

Supplemental Figure 7. Specific binding of FOS and JUN in promoters and enhancers with differential chromatin accessibility. (A) DNA

binding-site motifs bound by FOS and JUN domain proteins respectively. **(B)** ATAC-seq and ChIP-seq (H3K4me3, H3K27ac, H3K4me1) signals around the center of JUN+/- and FOS+/- DARs. **(C)** IGV track view of ATAC-seq, ChIP-seq (H3K4me1, H3K27ac, H3K4me3, FOS and JUN) normalized density of two representative examples with FOS and JUN binding sites.

SUPPLEMENTAL TABLES

Supplemental Table 1. Detailed information of mothers and infants

IDs	Sex	Delivery	Maternal age (years)	Maternal height (cm)	Maternal weight (pre-pregnancy) (kg)	BMI (pre-pregnancy)	Maternal weight (at delivery) (kg)	BMI (at delivery)	Gestational age (weeks)	Birth weight (kg)	Birth length (cm)
SGA1	Female	Cesarean	31	155	40	16.64932362	49.5	20.60353798	40.6	2.19	46
SGA2	Female	Vaginal delivery	27	163	49	18.44254582	67	25.21735858	36.1	1.93	43
SGA3	Female	Vaginal delivery	24	155	50	20.81165453	73.5	30.59313215	37.7	1.9	40
SGA4	Male	Vaginal delivery	28	159	54.5	21.55769155	63	24.91990032	39	2.57	49
SGA5	Female	Vaginal delivery	25	158	43	17.22480372	51	20.42941836	38.1	2.43	44
SGA6	Male	Cesarean	28	168	60.1	21.29393424	74	26.21882086	39.4	2.94	47
SGA7	Female	Cesarean	31	166	62	22.4996371	50	18.14486863	38.6	2.77	48
SGA8	Male	Cesarean	28	168	64.5	22.85289116	52.3	18.5303288	39.1	2.82	49
SGA9	Male	Cesarean	27	158	52	20.82999519	40	16.02307323	38	2.51	48
SGA10	Female	Cesarean	34	158	60	24.03460984	48	19.22768787	41.1	2.62	46
SGA11	Male	Cesarean	31	158	60	24.03460984	48	19.22768787	40	2.63	47
SGA12	Female	Vaginal delivery	31	150	58.5	26	47	20.88888889	38.4	2.25	46
AGA1	Female	Cesarean	29	158	48.8	19.54814934	60	24.03460984	40.3	3.4	49
AGA2	Male	Vaginal delivery	31	162	64.5	24.57704618	72.5	27.62536199	37.3	3.48	50
AGA3	Male	Vaginal delivery	28	163	83.9	31.57815499	91.9	34.58918288	40	3.21	50
AGA4	Male	Cesarean	35	160	50	19.53125	62.5	24.4140625	40.9	3.79	53
AGA5	Male	Cesarean	31	162	71.5	27.24432251	51.5	19.623533	37.1	2.9	49
AGA6	Female	Cesarean	31	158	58	23.23345618	46	18.42653421	39.1	3.19	50
AGA7	Male	Cesarean	30	158	75	30.0432623	60	24.03460984	39.6	3.26	49
AGA8	Female	Cesarean	27	162	73	27.81588173	58	22.10028959	40.1	2.9	48
AGA9	Female	Cesarean	29	165	71.7	26.33608815	55.7	20.45913682	40.4	3.3	49

Supplemental Table 2. Clinical Statistic characteristics of mothers and infants

Characteristics	AGA (n=9)	SGA (n=12)	*P*-values
**Mothers**			
Age(years)	30.11±0.77	28.75±0.83	0.261
BMI (Pre-pregnancy) (kg/m^3)	23.92±1.63	21.66±1.21	0.27
BMI (at pregnancy) (kg/m^3)	25.54±1.41	21.35±0.81	0.013*
Preeclampsia, n	0	1	/
**Infants at birth**			
Delivery (C/V)	7/2	7/5	/
Apgar at 5 min	10	10	/
Gender(F/M)	5/4	5/7	/
Gestational age (weeks)	39.42±0.45	38.84±0.39	0.924
Birth weight (kg)	3.47±0.12	2.15±0.16	0.000****
Birth weight (percentile)	10-90	<10	/
Birth length (cm)	49.67±0.47	46.08±0.76	0.002**

Datas are expressed as mean ± SEM and analyzed by two-tailed umpaired Student's t test. F: female; M: male; C: cesarean; V: vaginal delivery.

Supplemental Table 3. Fold changes of key genes regulated by CD44 in SGA

	Fold Changes in SGA vs. AGA	Fold Changes in SGA-si*CD44* vs. SGA-siControl
TNFSF4	3.48	0.47
CD44	3.30	0.33
SULF2	2.57	0.62
AADACP1	2.36	0.62
HSD17B2	1.98	0.50
TNFSF18	1.88	0.49
RNF182	1.80	0.64
CASP4LP	1.61	0.62
TFPI2	1.54	0.60
MLLT11	1.51	0.44
LYVE1	1.51	0.59
VCAM1	0.66	1.70
ADAMTS18	0.66	1.62
ITPR2	0.65	1.72
CNTNAP1	0.60	1.53
HAPLN1	0.59	1.77
GCKR	0.56	1.71
CROCCP3	0.56	3.12
HIF3A	0.51	2.24
IGFBP3	0.50	1.98
NPR1	0.46	1.75

Supplemental Table 4. List of top 10 motifs enriched in DARs

Class	Rank	Name	q-value (Benjamini)	Number of Target Sequences with Motif	Percent of Targets Sequences with Motif
**SGA-gained**	1	Fra1(bZIP)/BT549-Fra1-ChIP-Seq(GSE46166)/Homer	0	217	32.63
**SGA-gained**	2	Fos(bZIP)/TSC-Fos-ChIP-Seq(GSE110950)/Homer	0	222	33.38
**SGA-gained**	3	Atf3(bZIP)/GBM-ATF3-ChIP-Seq(GSE33912)/Homer	0	232	34.89
**SGA-gained**	4	JunB(bZIP)/DendriticCells-Junb-ChIP-Seq(GSE36099)/Homer	0	207	31.13
**SGA-gained**	5	Fra2(bZIP)/Striatum-Fra2-ChIP-Seq(GSE43429)/Homer	0	191	28.72
**SGA-gained**	6	BATF(bZIP)/Th17-BATF-ChIP-Seq(GSE39756)/Homer	0	225	33.83
**SGA-gained**	7	AP-1(bZIP)/ThioMac-PU.1-ChIP-Seq(GSE21512)/Homer	0	231	34.74
**SGA-gained**	8	Fosl2(bZIP)/3T3L1-Fosl2-ChIP-Seq(GSE56872)/Homer	0	141	21.2
**SGA-gained**	9	Jun-AP1(bZIP)/K562-cJun-ChIP-Seq(GSE31477)/Homer	0	106	15.94
**SGA-gained**	10	Bach2(bZIP)/OCILy7-Bach2-ChIP-Seq(GSE44420)/Homer	0	59	8.87
**SGA-lost**	1	Fra1(bZIP)/BT549-Fra1-ChIP-Seq(GSE46166)/Homer	0	116	21.56
**SGA-lost**	2	JunB(bZIP)/DendriticCells-Junb-ChIP-Seq(GSE36099)/Homer	0	116	21.56
**SGA-lost**	3	Fos(bZIP)/TSC-Fos-ChIP-Seq(GSE110950)/Homer	0	120	22.3

**SGA-lost**	4	BATF(bZIP)/Th17-BATF-ChIP-Seq(GSE39756)/Homer	0	127	23.61
**SGA-lost**	5	Atf3(bZIP)/GBM-ATF3-ChIP-Seq(GSE33912)/Homer	0	127	23.61
**SGA-lost**	6	AP-1(bZIP)/ThioMac-PU.1-ChIP-Seq(GSE21512)/Homer	0	133	24.72
**SGA-lost**	7	Fra2(bZIP)/Striatum-Fra2-ChIP-Seq(GSE43429)/Homer	0	102	18.96
**SGA-lost**	8	Fosl2(bZIP)/3T3L1-Fosl2-ChIP-Seq(GSE56872)/Homer	0	76	14.13
**SGA-lost**	9	Jun-AP1(bZIP)/K562-cJun-ChIP-Seq(GSE31477)/Homer	0	62	11.52
**SGA-lost**	10	ETS1(ETS)/Jurkat-ETS1-ChIP-Seq(GSE17954)/Homer	0	125	23.23

Supplemental Table 5. Primers used in this study

Type	Name	Sequence (5'-3')	Genomic Coordinates
**RT-qPCR**	RT-qPCR-GAPDH-F	TTGGTATCGTGGAAGGACTCA	
	RT-qPCR-GAPDH-R	TGTCATCATATTTGGCAGGTT	
	RT-qPCR-CXCL8-F	CTCCAAACCTTTCCACCCCA	
	RT-qPCR-CXCL8-R	TTCTCAGCCCTCTTCAAAAACT	
	RT-qPCR-IL1A-F	CATTGGCGTTTGAGTCAGCA	
	RT-qPCR-IL1A-R	CATGGAGTGGGCCATAGCTT	
	RT-qPCR-TNFSF18-F	CAATGGGTAGTCCCCTGCAT	
	RT-qPCR-TNFSF18-R	CACCTTGGGCTAAAGGGGAA	
	RT-qPCR-CD44-F	GCAGCCTCAGCTCATACCAG	
	RT-qPCR-CD44-R	GCTTGATGACCTCGTCCCAT	
	RT-qPCR-FOXF-F	TCTCGCTCAACGAGTGCTTC	
	RT-qPCR-FOXF-R	GTTCATCATGCTGTACATGGGC	
	RT-qPCR-COL3A1-F	CGCCCTCCTAATGGTCAAGG	
	RT-qPCR-COL3A1-R	TTCTGAGGACCAGTAGGGCA	
	RT-qPCR-IGFBP3 F	GAGAGTCAGCCTCCACATTC	
	RT-qPCR-IGFBP3 R	GCAAGCCATTCCTCCTTCCT	
	RT-qPCR-SFRP1-F	ATGAGTGCCCCACCTTTCAG	
	RT-qPCR-SFRP1-R	AATGCTGCAAGAACAAGCCG	
	RT-qPCR-CDH11-F	CCGCTGACTTGTGAATGGGA	
	RT-qPCR-CDH11-R	CAGTGATTTCTGGGGCCCTT	
	RT-qPCR-SULF1-F	GGCTTGATCGGCAACTAGGA	
	RT-qPCR-SULF1-R	GTTCCTCATCTGCCCTGACC	
**siRNA and overexpression**	siRNA-CD44-sense	CGGAAGUGCUACUUCAGACAATT	
	siRNA-CD44-antisense	UUGUCUGAAGUAGCACUUCCGTT	
	siRNA-NC-sense	UUCUCCGAACGUGUCACGUTT	
	siRNA-NC-antisense	ACGUGACACGUUCGGAGAATT	
	overexpression-CD44-F	TCTAGAGCTAGCGAATTCATGGAC AAGTTTTGGTGGCAC	
	overexpression-CD44-R	CTTCGCGGCCGCGGATCCTTACACC CCAATCTTCATGT	
**CRISPR interference**	Guide target-CD44-promoter	TGGTGTCCGGAGCGAACGGA	Chr11:35,139,285-35,139,304
	Guide target-CD44-enhancer_01	TCAACGCTGAACCAACATGG	Chr11:35,166,598-35,166,617

	Guide target-CD44-enhancer_02	TCCTTGCACAGTTCTAGTGG	Chr11:35,174,270-35,174,289
	Guide target-CD44-enhancer_03	GTGGTATGGGACCCCCCACT	Chr11:35,214,875-35,214,894
**ChIP-qPCR**	ChIP-qPCR-CD44-Promoter-F	TTACAGCCTCAGCAGAGCAC	
	ChIP-qPCR-CD44-Promoter-R	AACAGTGACCTAAGACGGAG	
	ChIP-qPCR-CD44-E1-F	CAGGGCACATTCTGGAGGAA	
	ChIP-qPCR-CD44-E1-R	ATCACACTTGGTGGCCGAAT	
	ChIP-qPCR-CD44-E2-F	GGTACTCACTCACTTCCTTGG	
	ChIP-qPCR-CD44-E2-R	TGGAATCAGACAGAACTGGCA	
	ChIP-qPCR-CD44-E3-F	GGATCTGAATCAGATGGTGAG	
	ChIP-qPCR-CD44-E3-R	GTTTGAGAACCACTTCTCTGG	
**3C-qPCR**	GAPDH-qPCR-F	GGAGAAGCTGAGTCATGGGT	
	GAPDH-qPCR-R	AAGACGGAATGGGGAGAAGG	
	3C-qPCR-CD44-Promoter-F	AACTTCCGAGGCAGCCTCATTG	
	3C-qPCR-CD44-E1-R	GGAGACAGGGACAGTACTGTTC	
	3C-qPCR-CD44-E2-R	CTGAAATTCCAATAGAGAGC	
	3C-qPCR-CD44-E3-R	CAGTTATCCATTATGAAGGCACC	
