# Supplementary material for: Reshaping the chromatin landscape in HUVECs from small-for-gestational-age newborns
Source: JCI Insight. 2025 Apr 22;10(8):e186812. doi: 10.1172/jci.insight.186812 (PMC12038915; doi:10.1172/jci.insight.186812)

Full unedited blots for Figure 2J

CD44

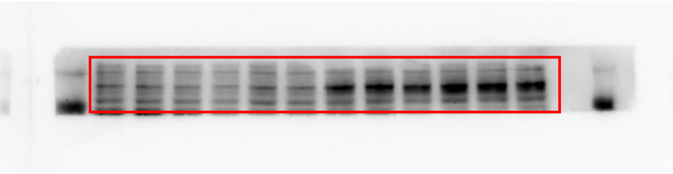

GAPDH

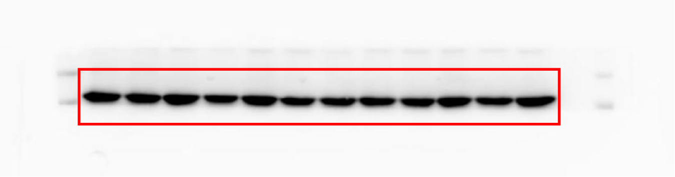

Full unedited blots for Figure 3K

p-ERK1/2

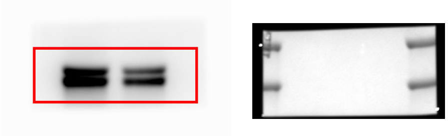

t-ERK1/2

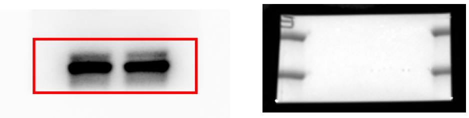

p-eNOS

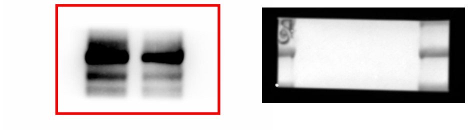

t-eNOS

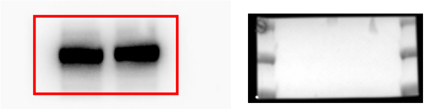

Full unedited blots for Figure 3L

p-ERK1/2

t-ERK1/2

Actin

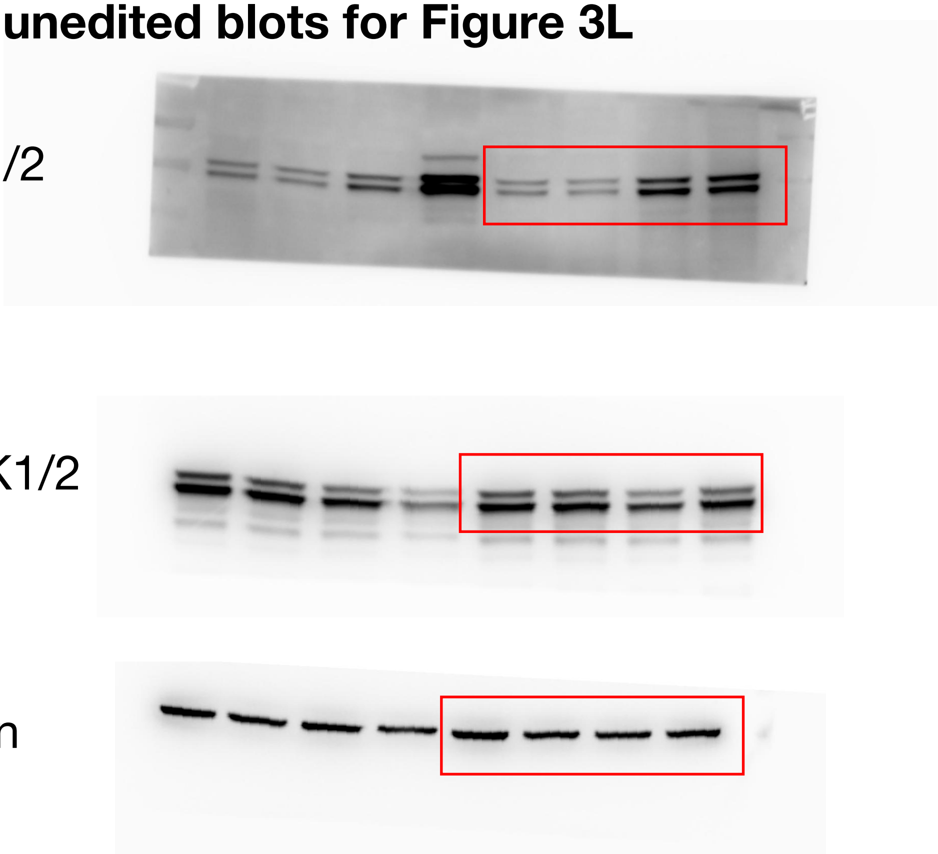

Full unedited blots for Figure 3M

p-eNOS

t-eNOS

Actin

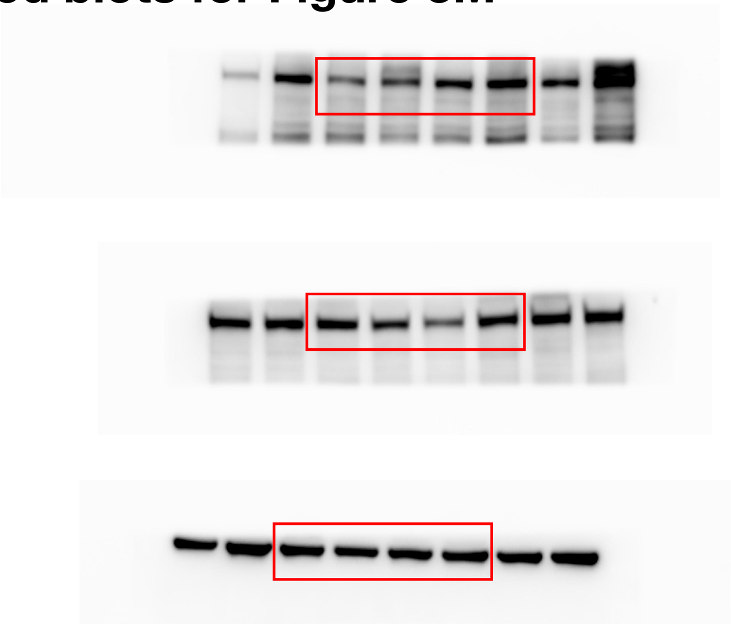

Full unedited blots for Supplemental Figure 4B

CD44

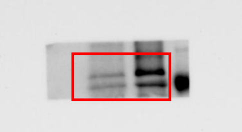

GAPDH

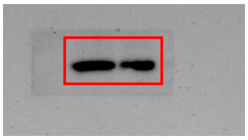

Full unedited blots for Supplemental Figure 4K

CD44

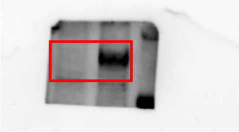

GAPDH

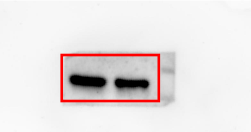

Full unedited blots for Supplemental Figure 6C

CD44

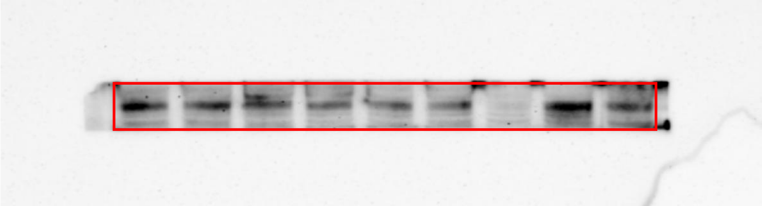

GAPDH

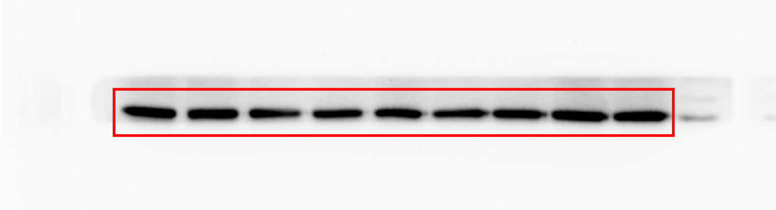

Supplement: Unedited blot and gel images [file jciinsight-10-186812-s009.pdf]
